# Genomic and Phenotypic Characterization of Two Novel Enterobacter Phages With EDTA‐Enhanced Antibiofilm Activity

**DOI:** 10.1002/mbo3.70396

**Published:** 2026-09-02

**Authors:** Kashif Haq, Zac Newland‐Smith, Martin Figgit, David Lee, Kwabena Duedu

**Affiliations:** ^1^ Department of Life Sciences, School of Health Sciences Birmingham City University Birmingham UK

## Abstract

Multidrug‐resistant members of the *Enterobacter cloacae complex* (ECC) are increasingly linked to difficult‐to‐treat infections and biofilm‐mediated antimicrobial tolerance. Here, two lytic phages, vB_EhoIP_HHH and vB_EluM_RZH, displaying podovirus‐like and myovirus‐like morphology, respectively, were isolated from the River Chelt. HHH has a 39,582 bp genome (51.2% GC, 63 ORFs), while RZH has a 174,197 bp genome (39.4% GC, 314 ORFs), with neither genome carrying antimicrobial resistance, virulence or lysogeny‐associated genes. VIRIDIC and VICTOR analyses placed HHH within Kayfunavirus and RZH within Karamvirus, supporting their classification as distinct species. Both phages demonstrated rapid adsorption, short latent periods and stability across physiological pH and temperature ranges. A phage cocktail targeting MDR ECC strain was evaluated with EDTA against established biofilms. Crystal violet assays showed the greatest biomass reduction at MOI 10 with 0.5–0.75 mM EDTA. Bliss independence analysis revealed localized synergy within this window but significant overall antagonism at higher EDTA concentrations. CFU enumeration confirmed greater activity against 24 h than 48 h biofilms. The optimized combination also reduced recoverable bacteria in a fibroblast infection model while maintaining low LDH release. These findings identify two novel lytic Enterobacter phages and support a narrow EDTA concentration window for enhanced phage‐mediated antibiofilm activity.

## Graphical abstract

Two novel lytic Enterobacter phages, HHH and RZH, displayed targeted antibiofilm activity against MDR Enterobacter cloacae complex biofilms when combined with EDTA. Activity was strongest within a narrow low‐millimolar EDTA window, reducing biofilm biomass and viable burden, while higher EDTA concentrations shifted the interaction towards antagonism.

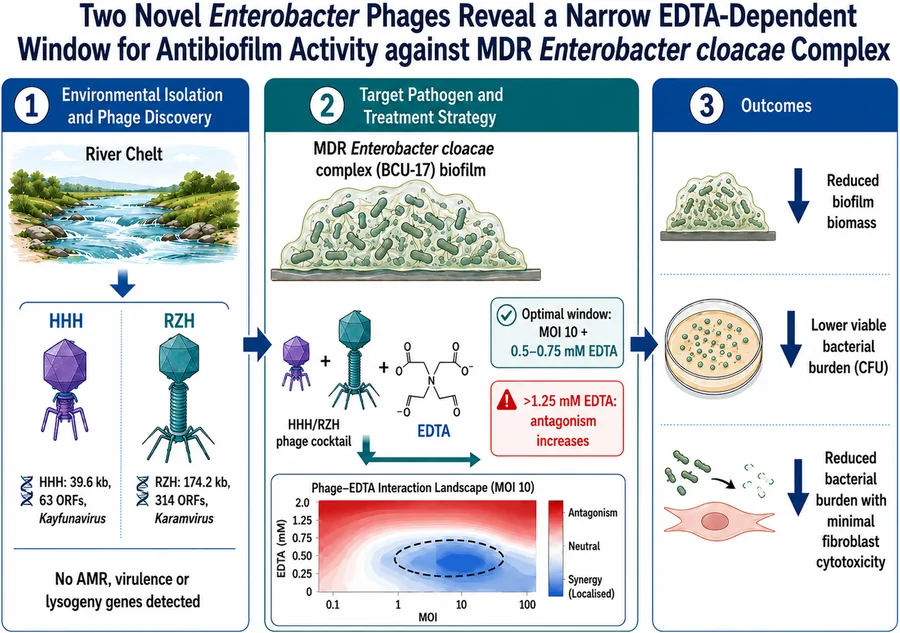

## 1 Introduction

The *Enterobacter cloacae complex* (ECC) has evolved as a major cause of nosocomial and community acquired infections, responsible for soft tissue infections, osteomyelitis, urinary tract infections, respiratory infections and septicemia (Annavajhala et al. 2019; Davin‐Regli et al. 2019; Girlich et al. 2021; Miltgen et al. 2021). From this group of Gram‐Negative pathogens, *E. hormaechei* and *E. ludwigii* have emerged as organisms of concern (Chen et al. 2022a; Baek et al. 2024). These pathogens usually demonstrate a multi drug resistant phenotype (Peirano et al. 2018) harbor extended spectrum β‐lactamase and carbapenemase genes, *bla*_KPC,_ *bla*_NDM_, *bla*_OXA,_ *bla*_vim_ and *bla*_IMP_ (Annavajhala et al. 2019; Chen et al. 2022a; Baek et al. 2024; Emeraud et al. 2022; Kizny Gordon et al. 2020). Moreover, chromosomal encoded class C β‐lactamases, the overexpression of efflux pump systems AcrAB‐TolC (Hu et al. 2022; Guérin et al. 2016) make these organisms difficult to treat with antibiotics.

Over the last decade, phage therapy has been used to treat such infections (Hitchcock et al. 2023). Moreover, it has been reported, emergence of phage‐resistant bacteria can hinder this process (Kortright et al. 2019), the use of phages in a cocktail can broaden the lytic range and reduce the rise of phage resistant populations, but studies have demonstrated they fail to fully inhibit the re‐emergence of bacterial resistant cells (Duc et al. 2020). Other approaches such as combining phages and antibiotics have been explored with greater success than either agent alone (Morris et al. 2025; Cui et al. 2024), however synergistic activity is decreased when biofilms are mature, if timing or dosing have not been optimized and whether there is an antagonistic relationship with the chosen antibiotic (Lee et al. 2025). Ethylenediaminetetraacetic acid (EDTA) is a well‐known chelating agent, widely used in healthcare, within pharmaceutical formulations and has antimicrobial properties. EDTA disrupts biofilm integrity by chelating divalent cations within the extracellular polymeric substance (EPS) and destabilizing its structure (Finnegan and Percival 2015).

Several studies have characterized phages against *E. cloacae* (Shymialevich et al. 2024; Cheng et al. 2023; González‐Gómez et al. 2024; Nasr‐Eldin et al. 2023; Cieślik et al. 2022; Chen et al. 2022b; Rahimzadeh Torabi et al. 2021; Chen et al. 2021; Wójcicki et al. 2021), to name a few. However, there are limited studies examining phage lytic behavior and antibiofilm activity specifically against *E. hormaechi* and *E. ludwigii* furthermore, very few studies have explored the use of EDTA and phages in a synergistic manner to tackle biofilms caused by the ECC. In this study, two new bacteriophages were isolated and characterized against multidrug resistant *E. hormaechi* and *E. ludwigii* clinical type strains, and their activity was further evaluated in combination with EDTA against biofilm biomass. The data presented will increase the knowledge base of lesser‐known phages against the *E. cloacae* complex and provide insight into their potential as antibiofilm agents.

## 2 Materials and Methods

### 2.1 Bacterial Strain and Culture Conditions

#### 2.1.1 Isolation, Purification and Host Range of Phage

Isolation and purification of phages were from the River Chelt (51.896062 N −2.069746 W), which recently had sewage pumped into it into from an overflow pipe. The sewage sample was centrifuged, 10,000 *g* for 10 min to remove debris, samples were then added to equal volumes of 2x Luria‐Bertani broth supplemented with 100 mM CaCl_2_ and 150 mM MgSO_4_. 100 µl of host bacteria; *Enterobacter hormaechei* (EH‐BCU 9) or *Enterobacter ludwigii* (EL‐BCU 10) were grown to exponential log phase and added to the suspension. Co‐culture was incubated at 37°C at 150 rpm for 18–22 h. The following day chloroform at 0.1 volume of the suspension was added and suspension was incubated at room temperature for 30 min with gentle rocking. The sample was centrifuged at 11,000 *g* for 5 min to remove bacteria and any debris. 6 mL were transferred into a 15 mL falcon tube and 1.5 mL of PEG/NaCl (5x) was added, the suspension was then incubated at 4°C for 24 h. The following day, the mixture was centrifuged at 10,000 x *g* for 20 min, supernatant was decanted, and the pellet was re‐suspended in 600 µL of SM buffer and filtered through a 0.22 µm membrane. Host range of phage was performed as previously described (Ngiam et al. 2021), by spot test using bacterial strains available. Briefly, 100 µL of log phase bacteria was cultured on to LB agar plates via the double overlay method. 10 µL of phage at 10^8^ PFU/mL was spotted onto the plate and incubated at 37°C for 24 h. Efficiency of plating (EOP) was determined for strains showing susceptibility by spot testing. Briefly, serial ten‐fold dilutions of each phage were plated on the propagation host and on each test, strain using the double‐layer agar method. Plaques were enumerated after incubation, and EOP was calculated as the ratio of phage titer on the test strain to phage titer on the corresponding propagation host (Ngiam et al. 2021). EOP values were calculated from three independent biological replicates and reported as mean ± SD.

#### 2.1.2 Transmission Electron Microscopy

Morphology of phage isolated was determined by transmission electron microscopy (Ackermann 2009) and conducted by electron microscopy suite, Open University (https://emsuite.stem.open.ac.uk/). Briefly, 10 µl of phage lysate at a concentration of 1 × 10^9^ PFU/ml was added onto a copper grid and negatively stained with 2% uranyl acetate. Image was processed through a JEOL JEM 1400 transmission electron microscope at a voltage of 120 kV.

#### 2.1.3 Optimal Multiplicity of Infection (MOI)

MOI was evaluated by adding phage to its host bacterium (10^7^ CFU/mL) at MOI's 0.01, 0.1, 1, 10, and 100. Cultures were incubated for 5 h at 37°C, centrifuged at 12,000 x g for 5 min, and supernatant was filtered through a 0.22 µm filter. Phage titer was determined by the double layer agar assay, MOI yielding the highest titer was used in the following experiments.

#### 2.1.4 One‐Step Growth Curve

This assay was performed to determine the latent period and burst size of phages vB_EhoIP_HHH and vB_EluM_RZH. The methodology was based on a previously described method (Kropinski 2018), with slight modifications. Briefly, host strains were grown at 37°C in LB broth to mid‐exponential phase, equivalent to an OD600 of approximately 0.4. Ten milliliters of bacterial culture was adjusted to 1 × 10^8^ CFU/mL and combined with the appropriate phage stock to produce a multiplicity of infection of 0.01, equivalent to an initial phage input of approximately 1 × 10^6^ PFU/mL. Phages were allowed to adsorb to host cells for 15 min at 37°C. The suspension was then centrifuged at 10,000 × g for 4 min to remove unadsorbed phages in the supernatant, and the infected bacterial pellet was resuspended in 10 mL of pre‐warmed LB broth supplemented with 1 mM CaCl_2_. Cultures were incubated at 37°C with shaking at 150 rpm for 80 min. Samples were collected every 10 min, and phage titers were determined using the double‐layer agar assay. Phage titers at each time point were expressed as PFU/mL and plotted against time to determine the latent period and burst size. The latent period was defined as the interval before the first sustained increase in extracellular phage titer. Burst size was calculated as the increase in phage titer between the pre‐burst and plateau phases divided by the estimated number of infected bacterial cells, using the formula: Burst size = (mean plateau PFU/mL − mean pre‐burst PFU/mL)/estimated infected cells/mL The mean plateau titer was calculated from the final three time points of the one‐step growth curve. The estimated number of infected cells was calculated from the initial phage input and the adsorption fraction determined during the adsorption step. Experiments were performed using three independent biological replicates, and results were presented as mean values from these replicates.

#### 2.1.5 Adsorption Assay

Phage adsorption kinetics of the isolated phages were determined at MOI 0.01 and calculated through the number of unadsorbed phages (Hyman and Abedon 2009). Mixture was incubated for 15 min at 37°C in a shaking incubator (150 rpm). 100 µl was removed every 5 min for 20 min and added to 900 µl ice cold LB broth, following centrifugation at 12,000 x *g* for 4 min, supernatant was used to determine the concentration of viruses through the overlay assay. Repeat sampling was performed every 5 min, and unadsorbed phages were calculated as a percentage. Adsorption rate was calculated using the following formulas *k*_*a*_ = (2.3/*Bt*) × log(*P*_*o*_/*P*), where *k*_*a*_ is the adsorption rate constant in ml/min, *B* is the concentration of bacterial cells, and *t* is the time interval in which the titer falls from the original population, *P*_*o*_, to the final population (Zurabov and Zhilenkov 2021).

#### 2.1.6 Influence of pH and Temperature on Phage Stability

Stability of phages in harsh environments was evaluated through the effects of pH by subjecting a known concertation of phages (10^8^ PFU/mL^−1^), into 1 mL SM buffer with pH varying from 1 to 13. Acidity and alkalinity levels were adjusted with 1 M NaOH and 1 M HCL, samples were incubated for 1 h at 37°C. Thermal stability was assessed using 500 µL of 10^8^ PFU/mL^−1^ stock and incubating at temperatures between 4°C and 65°C for 60 min. Phage titers were determined through the double overlay assay and expressed as percentage of phages survived, assay was run in triplicates.

#### 2.1.7 Bacterial Lysis Assay

Host bacteria were grown to mid log phase in LB broth, they were then centrifuged, and pellets were resuspended in LB broth, to obtain a bacterial concentration 1 × 10^8^ CFU/mL. Phage MOI's at 100, 10, 1, 0.1, and 0.01 were added to bacterial suspensions. Negative control consisted of SM buffer and positive control consisted of host bacteria. Plate was incubated at 37°C in plate reader with intermittent shaking, absorbance at 0D6_600nm_ every 30 min for 8 h. Lysis curves were obtained by plotting OD against time.

#### 2.1.8 Biofilm Eradication Assay Using Phage Cocktail and EDTA in a Checkerboard Assay Format

Although phage isolation and biological characterization were performed using the original propagation hosts, *E. hormaechei* BCU‐9 and *E. ludwigii* BCU‐10, MDR ECC strain BCU‐17 was selected for biofilm assays because it was susceptible to both vB_EhoIP_HHH and vB_EluM_RZH. This enabled the HHH/RZH cocktail to be assessed against a single shared biofilm‐forming host. Evaluation of biofilm biomass reduction was conducted using previously known methods (Adnan et al. 2020), with slight modifications. Briefly, fresh bacterial suspensions of host strains were grown in Luria‐Bertani broth and incubated at 37°C overnight. Following day, cultures were centrifuged at 4000 x *g* for 10 min, the supernatant was discarded and pellets were washed twice and in 1X PBS. and resuspended in Luria‐Bertani broth. Density was adjusted to 0.5 McFarland (1 × 10^8^ CFU/mL) and further diluted 1:100 (1 × 10^6^ CFU/mL). 200 µL was dispensed into a 96 well plate. Controls consisted of media only and blank wells, plate was incubated at 37°C overnight. After incubation, planktonic cells were aspirated, and the plate was washed three times with distilled water. 50 µL EDTA at final concentrations of 0.5, 0.75, 1, 1.25, 1.50, 1.75, 2.0 and 2.25 mM and phage cocktail titers of MOI of 0.01 – 100, based on initial inoculum were dispensed into their respective rows and columns (Fig S1). Plate was incubated for 24 h at 37°C. Following incubation, contents of the plate were aspirated, and plate was washed as before., it was fixed with methanol and stained with 0.1% crystal violet. After 20 incubating for 20 min at room temperature excess stain was decanted, and 33% glacial acetic acid was added to solubilize the dye. Contents were transferred to a separate plate, and optical density was read at 570 nm. Biofilm percentage reduction was calculated by normalizing the data (Liu et al. 2020) and SynergyFinder+ (Zheng et al. 2022) was used to assess synergy between EDTA and the phage cocktail via the Bliss independence model. Synergy scores were calculated across the full checkerboard matrix rather than by pairwise comparison of individual wells. Positive Bliss scores indicated synergy, scores close to zero indicated additivity, and negative scores indicated antagonism. Statistical significance was determined by SynergyFinder+ using replicate variability across the complete dose–response surface. Quantification of bacteria within the biomass was determined for selective combinations via scraping the biofilm into a sterile 1.5 mL microcentrifuge tubes containing 1x PBS. Tubes were centrifuged at 10,000 x *g* for 5 min; the supernatant was decanted and pellets were washed 1x PBS and left to air dry for 10 min. Pellets were‐suspended in 100 µL 1x PBS, serial diluted and plated on LB agar. Results were expressed as Log_10_ (Stepanović et al. 2000). Process was repeated for 48 h biofilms. All data produced was from three biological repeats.

#### 2.1.9 Phage—EDTA Toxicity to Fibroblasts and Their Activity against Host Bacteria

A commercial Lactate dehydrogenase (LDH) assay (CytoTox 96 Promega) was used to assess cytotoxicity towards human dermal fibroblasts. Fibroblasts were seeded at a density of 1 × 10^4^ cells/mL (150 µL per well) in a 96 well tissue culture plate and incubated at 37°C with 95% air and 5% CO_2_ in Dulbecco's Modified Eagle Medium (DMEM) with 10% fetal bovine serum. Overnight bacterial culture was grown to exponential phase at 37°C, with shaking at 150 rpm. Bacterial cells were harvested by centrifugation (10,000 x *g*, 4°C, 90 s), washed twice with PBS to remove residual metabolites and media, they were then resuspended in DMEM. 100 µl of bacterial suspension at 1 × 10^6^ CFU/mL was added to the plate, EDTA at 0.75 mM and phage cocktail at MOI 10 were also dispensed into the appropriate well and plate was incubated for 2 and 4 h. LDH release was measured via the manufactures protocol, with minor modifications, briefly After incubation, 50 µL of supernatant from each well was transferred into a sterile 96 well microtitre plate and 50 µL of LDH substrate mix was added to each well. Plate was incubated for 30 min in the dark at room temperature. Reaction was stopped by adding 50 µL of stop solution and absorbance read at 490 nm, and results were expressed as percentage of LDH released (Kosznik‐Kwaśnicka et al. 2023). Activity of phages in bacterial infected fibroblast culture was assessed in a similar manner, however after the inoculation of bacteria, and incubation of 2 h, phages HHH/RZH at MOI 10 and EDTA (0.75 mM) was added to the appropriate wells. Untreated wells were considered as a control; the plate was incubated for a further 2 and 4 h. At each time point, 50 µL of the supernatant was collected, serially diluted in distilled water and 50 µL of each dilution was inoculated onto a LB agar. Plates were incubated for at 37°C for 24 h, and viable bacterial counts were determined by colony‐forming unit (CFU) enumeration (Kosznik‐Kwaśnicka et al. 2023; Shan et al. 2018).

#### 2.1.10 DNA Extraction, Sequencing and Annotation

DNA was extracted using the NORGEN BIOTEK phage DNA isolation kit and following its protocol. Nano Drop was used to quantify DNA. Phage lysate at PFU/mL of 10^9^ was treated by adding 100 µL of DNase I 10x buffer, 1 µl DNase I (1U/µL) and 1 µL RNase A (10 mg/mL). The lysate was then incubated at 37°C for 90 min. DNase I and RNase A activity was inhibited by adding 20 µL 0.5 M EDTA. Phage protein capsid was digested by adding 1.25 µL Proteinase K (20 mg/mL), and incubated for another 90 min at 56°C.

Samples were sequenced by microbesNG (https://microbesng.com), genomic DNA libraries were prepared via the Nextera XT Library Prep Kit, following the manufactures protocol, Library preparation and DNA quantification was performed on the Hamilton MicrolaB STAR automated handling system. The libraries were sequenced on Illumina NovaSeq. 6000 using a 250 bp paired end protocol. Raw reads were trimmed using Uincycler, SPAdes were used to assembly data, and Pilon for polishing through BV‐BRC genome annotation service and RAST (Wattam et al. 2017; Brettin et al. 2015). Function of CDS were confirmed manually using BLASTp (BLAST: Basic Local Alignment Search Tool) against non‐redundant protein sequences (E < 10^−5^). Closely related phages were identified via BLASTn (BLAST: Basic Local Alignment Search Tool) and whole genome phylogenetic analysis between the phages were analyzed using the Virus Classification and Tree building online tool (VICTOR) (Meier‐Kolthoff and Göker 2017) Genome via BLAST Distance Phylogeny approach (GBDP), were the algorithm “coverage,” distance formulae *d*_*5*_, and confidence levels were calculated using the recommended settings of GGDC (https://ggdc.dsmz.de/victor.php). VIRDIC, (Virus Intergenomic Distance Calculator) was used to determine taxonomic classification and intergenomic distance between the phages using BLASTn and calculating pairwise average nucleotide identity (Moraru et al. 2020). NGPhylogeney.fr (Lemoine et al. 2019) was used to determine the evolutionary relationship between the closely related phages. The genomic map of phages was visualized through Proksee (Grant et al. 2023).

#### 2.1.11 Nucleotide Sequence Accession Numbers

Assembled data of bacterial strains *Enterobacter hormaechei* and *Enterobacter ludwigii* were deposited in the National Centre for Biotechnology Information (NCBI) database under the BioProject accession number PRJNA1129045 and PRNJA1354818; meta data can be found in the Sequence Read Archive (SRA) SUB14455581 and SUB15423642

The complete genome sequence of the phages was deposited in GenBank under the name Enterobacter phage vB_EholP_HHH, complete genome; accession number PQ122694.1 and Enterobacter phage vB_EluM_RZH; accession number PQ140450.1. Going forward, vB_EholP_HHH and vB_EluM_RZH will be known as phage HHH and RZH, respectively.

### 2.2 Statistical Analysis

All experiments were performed using three independent biological replicates. Heatmap data represent the mean percentage reduction in biofilm biomass calculated from three biological replicates. Growth curves and bar graphs are presented as mean ± standard deviation (SD). Statistical significance was assessed using one‐way analysis of variance (ANOVA) followed by Dunnett's multiple comparisons test, with treatment groups compared against the relevant untreated control, using XLSTAT in Microsoft Excel. A *p* value < 0.05 was considered statistically significant.

## 3 Results and Discussion

### 3.1 Isolation and Morphology

Antibiotic sensitivity data, host strain metadata and supporting genomic data are provided in Supporting Information S1: Table S1–S5. Briefly, MDR ECC isolates harbored extended ‐spectrum β‐ lactamases, carbapenemase and intrinsic resistance associated mechanisms.

**Figure 1:**
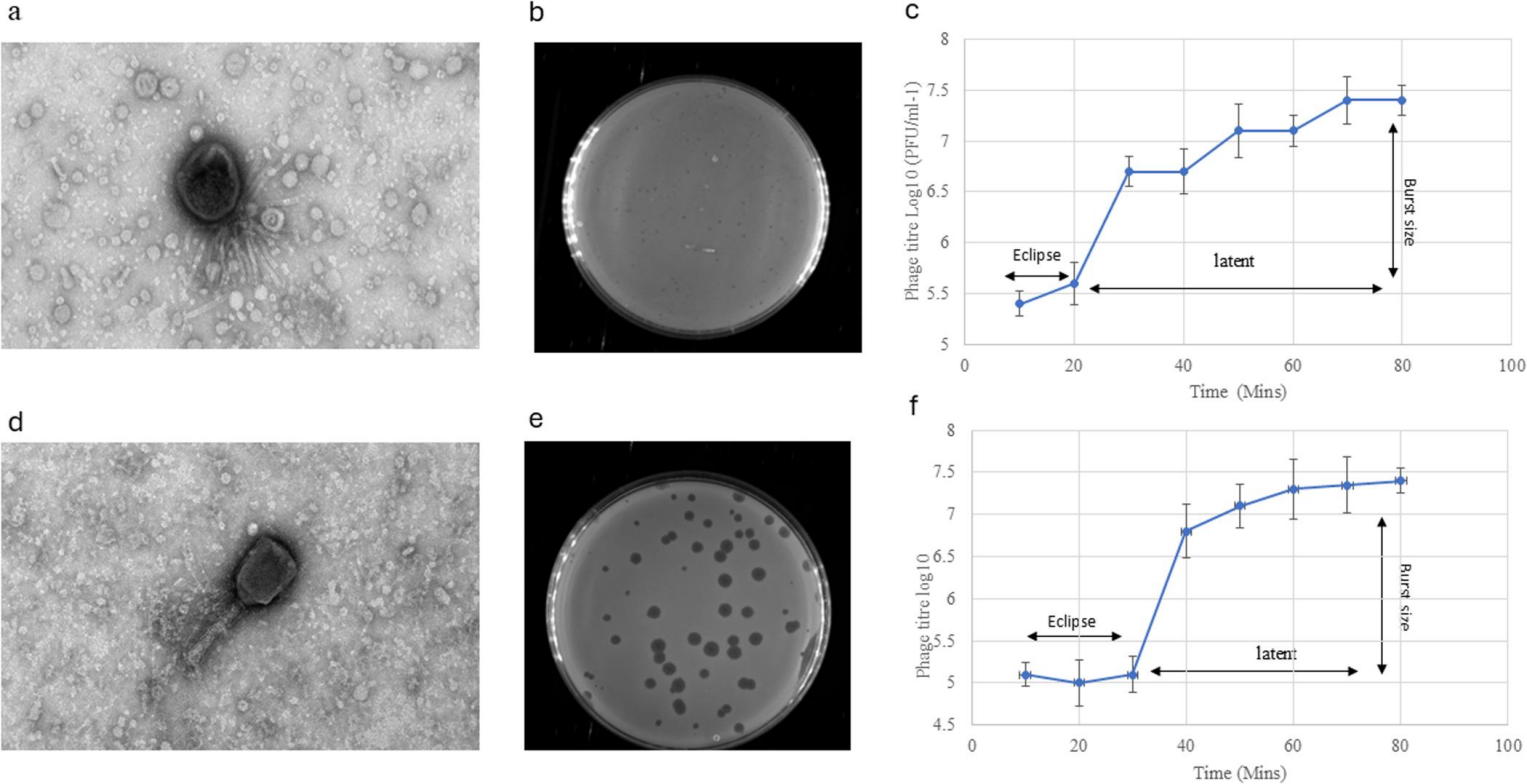
Representative transmission electron micrographs and plaque morphology of bacteriophages HHH and RZH, together with one‐step growth curves. (a) Transmission electron micrograph of phage HHH, showing morphology consistent with a podovirus‐like phage, corresponding to the former family Podoviridae, with an icosahedral head and short non‐contractile tail. HHH had an estimated head width of 39 ± 5 nm, head length of 42 ± 5 nm, and tail length of 12 ± 5 nm. (b) Plaque morphology of HHH, showing clear plaques consistent with lytic activity. (c) One‐step growth curve of HHH, showing an eclipse phase followed by a latent period and phage release, with an estimated burst size of 60.8 virions per infected bacterial cell. (d) Transmission electron micrograph of phage RZH, showing myovirus‐like morphology with an icosahedral head and long contractile tail. RZH had an estimated head width of 47 ± 5 nm, head length of 52 ± 5 nm, and tail length of 73 ± 5 nm. (e) Plaque morphology of RZH, showing clear lytic plaques. (f) One‐step growth curve of RZH, demonstrating phage replication kinetics and an estimated burst size of 73.5 virions per infected bacterial cell. Data in one‐step growth curves are presented as mean ± SD.

Both phages were isolated after propagation with their respected hosts. HHH and RZH produced clear plaques with diameters of 0.50 ± 2.0 mm (Figure 1a). Transmission electron microscopy (Figure 1b) revealed phage HHH belonged to the *Autographiviridae* family, and its morphology corresponds to the former family of *Podiviridae*, with a non‐contractile tail. Head width of 39 ± 5 nm, head length of 42 ± 5 nm and its tail length was 12 ± 5 nm. Phage RZH displayed characteristics of a myovirus, with a head width of 47 ± 5 nm, head length of 52 ± 5 nm and tail length of 73 ± 5 nm.

#### 3.1.1 Genomic Characterization of Phages

Whole genomic sequencing of both phages was undertaking using Illumina platform. Phage HHH is composed of linear ds DNA, with a length of 39,582 bp and GC Content 51.2%. Phage RZH is a linear ds DNA virus with a length of 174,197 bp and GC content 39.4. No virulence genes or antimicrobial resistant genes were found within both genomes, analysis by PhageLead (Yukgehnaish et al. 2022) revealed no genes related to temperate phages, furthermore, RAST and BV‐BRC (Wattam et al. 2017) predicted Phage HHH had 63 ORFs, 24 were annotated with known functions, whereas 39 were hypothetical proteins. In contrast, phage RZH had 314 CDS, 114 with known functions and 200 with unknown. No tRNA were detected in phage HHH using ARAGORN (Laslett. 2004), however. codon‐usage analysis of phage RZH revealed a number of codons AAA, GAA, GAC, AAC, ATG, and CAA, each of which is matched by a cognate phage‐encoded tRNA. This concordance between codon demand and tRNA's seems to be consistent with adaptive translation and may support overcoming host translational constraints, such as countering host defense systems, expanding host range and sustaining late‐stage replication (Lomeli‐Ortega and Balcázar 2024).

Annotated proteins with known or predicted functions were categorized into functional modules in Supporting Information: Table S6 and S7. Phage RZH encoded a large and functionally complex genome, with numerous predicted proteins associated with nucleotide metabolism, DNA replication, recombination, transcriptional regulation, structural assembly, and lysis (as detailed in Supporting Information: Table S7). For example, several genes characteristic of T4‐like myophages, including rIIA, rIIB, lysis inhibition‐associated proteins, nucleoid disruption protein Ndd, DNA topoisomerase, DNA helicases, DNA polymerase, ribonucleotide reductase subunits, thymidylate synthase, dCMP deaminase, and multiple endonucleases (Petrov et al. 2010). Of note, phage RZH also encoded a broad collection of structural proteins, involved in host recognition and adsorption (Zinke et al. 2022), including long tail fiber proteins (ORF 24, ORF 26–28, ORF 93, 121, 123, 126), baseplate hub proteins (ORF 94, 96–99) baseplate wedge subunits (ORF 95, 97, 100, 131–136, 140, 141, 180), neck protein (ORF 127–128), capsid and scaffolding associated proteins (ORF 107, 115, 116, 308, ORF 114–120) (Zinke et al. 2022). The lysis module of phage RZH consisted of a holin (ORF 23), a putative spanin inner membrane subunit (ORF 57), numerous proteins involved in lysis inhibition (rIIA, ORF 1), coordinating lysis timing (rIIB, ORF 2), a lysis inhibition accessory protein (ORF 75) and a lysis inhibition regulator membrane protein (ORF 200). Additionally, ORF 180 encodes a baseplate hub/tail lysozyme, which most likely acts as a virion associated peptidoglycan hydrolase as the baseplate hub lysozyme is part of the tail structure, that is involved in the initial infection process whereas endolysins are produced inside the infected bacterial cell to degrade the peptidoglycan membrane from within and occurs in the late stages of the infection cycle (Rodríguez‐Rubio et al. 2012). Notably, no clearly annotated soluble endolysin was identified in the RZH genome. Transmembrane topology prediction using TOPCONS (Bernsel et al. 2009) and DeepTMHMM (Hallgren et al. 2022) indicated that ORF73, annotated as a hypothetical protein, lacks predicted transmembrane domains, and SignalP (Teufel et al. 2022) did not detect a signal peptide (Figure S1). ORF73 is located near CDS75, which encodes a lysis inhibition accessory protein, suggesting that this region may contribute to the broader lysis module. Although these features are consistent with a soluble, non‐membrane‐associated protein a familiar trait with T4‐like phages (Schwarzkopf et al. 2025), ORF73 cannot be assigned as an endolysin without additional conserved‐domain or functional evidence. It should therefore be considered a candidate lysis‐associated protein pending further analysis.

**Figure 2:**
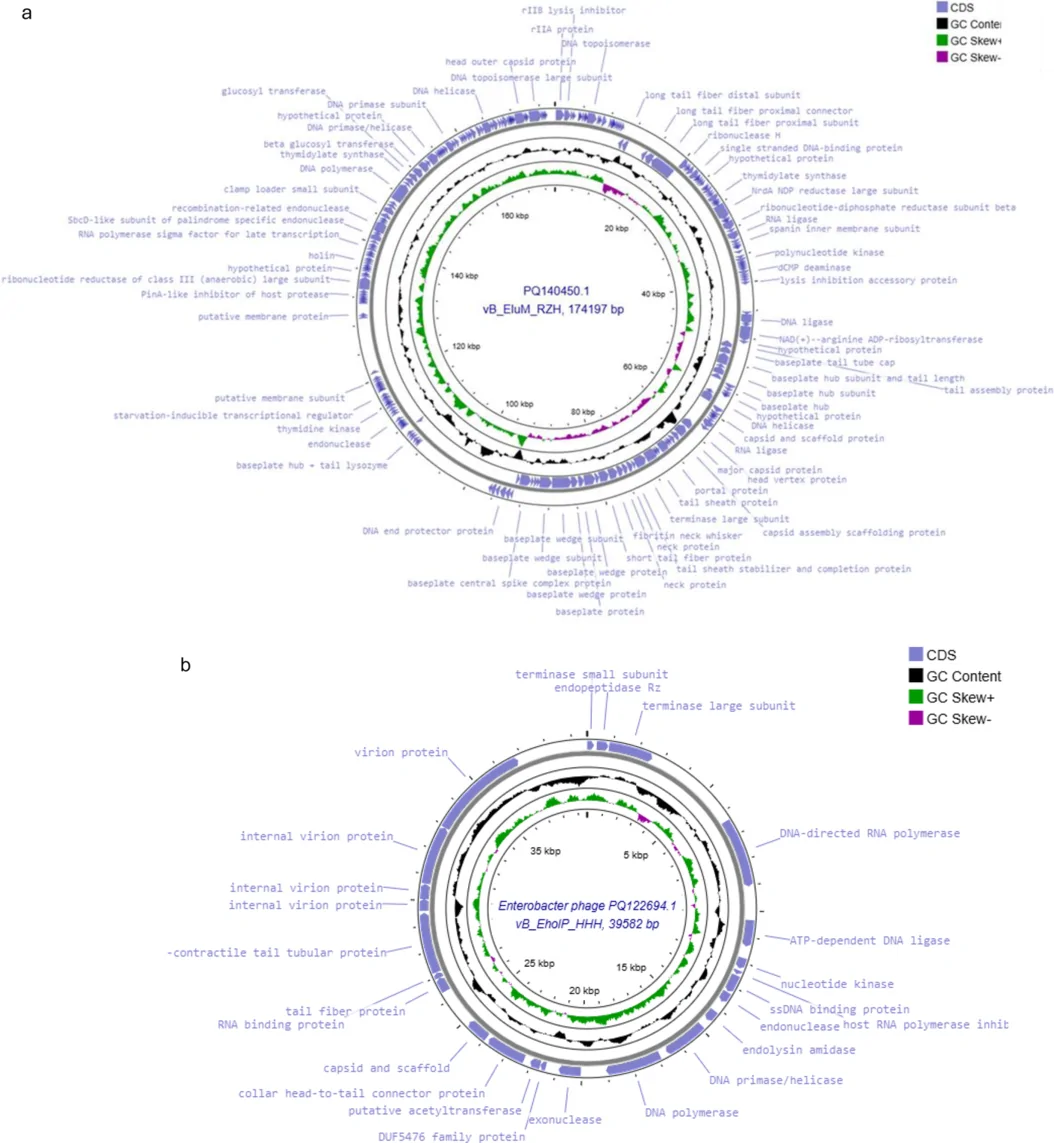
Circular genome map representation of the double‐stranded DNA genome of Enterobacter phage vB_EluM_RZH (PQ140450.1), comprising 174,197 bp. Predicted coding sequences are shown as blue arrows and are arranged according to their genomic position and transcriptional orientation. (b) Circular genome map representation of the double‐stranded DNA genome of Enterobacter phage vB_EhoIP_HHH (PQ122694.1), comprising 39,582 bp. Predicted coding sequences are shown as blue arrows and are arranged according to genomic position and transcriptional orientation.

Phage HHH possessed a smaller genome, but still encoded functional modules (Supporting Information: Table S6), such as the packaging module, consisting of small and large terminase subunits (ORF 1 & 3), which are required for assembling new viral particles (Fokine and Rossmann 2014). Furthermore, the replication module encoded a range of proteins involved in DNA replication and metabolism, including, DNA ligase (CDS 26), nucleotide kinase (CDS 29), host RNA polymerase inhibitor (CDS 31), ssDNA binding protein (CDS 32), DNA (primase/helicase (CDS 37), DNA polymerase (CDS 39), exonuclease (CDS 44), RNA binding protein (CDS 57). Of note, the genome also encodes a DNA‐Directed RNA polymerase, a hallmark feature of T7‐like phages, enabling transcription without the use of the host RNA polymerase (Cuervo et al. 2013). Collar head to tail connector protein (CDS 50), capsid and scaffold (CDS 51), tail fiber protein (CDS 56) and the non‐contractile tail tubular protein (CDS58) all contributed to the structural module and hall characteristics of T‐7 like phages (Cuervo et al. 2013). The lysis module consisted of a spanin component (endopeptidase Rz, CDS 2), part of a specialized lysis system that disrupt the outer membrane and bridging the inner and outer membranes of target bacteria, it usually functions in final step lysis following the holin‐endolysin mediation of peptidoglycan disruption (Fernandes and São‐José 2018). The endolysin amidase (CDS), is a small single protein that does not have a cell wall binding domain, allowing it to degrade bacterial peptidoglycan of different Gram‐negative bacteria (Sharma et al. 2016). Four internal virion proteins were identified (CDS 59–62), Topology and signal peptide analysis of HHH ORF59–ORF61 (Figure S2‐S4) suggests that these proteins are unlikely to function as membrane‐anchored lysis proteins. TOPCONS and DeepTMHMM predicted no transmembrane helices for ORF59, ORF60 ORF61 or ORF 62, with DeepTMHMM classifying each as globular rather than membrane associated. SignalP 6.0 also did not detect classical signal peptides in ORF 59, ORF 61 and ORF 62, supporting their interpretation as soluble, non‐membrane‐associated proteins. In contrast, ORF60 (Figure S5) contained a predicted N‐terminal Sec/SPI signal peptide despite lacking transmembrane domains, suggesting that it may be exported and processed as a soluble periplasmic protein. Given that ORF59–ORF61 are located within the predicted internal virion protein region of the HHH genome, these proteins are more likely to be associated with virion structure, host cell entry, packaging genome delivery or early infection processes than with holin/spanin‐mediated lysis. However, because conserved‐domain and functional evidence is currently limited, ORF 59–ORF 62 should be regarded as putative soluble virion‐associated proteins pending further validation. Of note, ORF 62 showed strong similarity to protein XTB81134.1 from Escherichia phage BUCT788, annotated as a peptidoglycan transglycosylase. This suggests that ORF62 may encode a soluble peptidoglycan‐degrading enzyme associated with local cell wall remodeling during infection (Sharma et al. 2016), however further conserved‐domain analysis and functional validation is required (Figure 2).

Specialty databases within the BV‐BRC pipeline, including VFDB, TCD, Victors, CARD and NDARO, detected no antimicrobial resistance, toxin‐related or virulence‐associated genes in either genome. In addition, PHASTEST (Wishart et al. 2023) detected no integrase, repressor or other prophage‐associated lysogeny markers, supporting the PhageLead safety assessment.

#### 3.1.2 Phylogenetic Analysis

BLASTn analysis identified eleven closely related phages to vB_EluM_RZH, all infecting *Enterobacter* species. The highest similarity was observed with phage vB_ECC_CW742 (PV019367.1), which exhibited 94% query coverage and 94.9% nucleotide identity. The lowest similarity among the selected phages was observed with vB_Ecl_id4496 (PQ824230.1), showing 94% query coverage and 88.97% identity. Overall, nucleotide identities ranged from 89% − 95%, indicating close but not identical genomic relationships. To further investigate evolutionary relationships, a whole‐genome phylogenetic tree was constructed using VICTOR and visualized in iTOL (Figure 4a). The resulting tree showed phage RZH clusters within a well‐supported clade of Enterobacter‐infecting phages, sharing an ancestorial linage with phage vB_ECC_CW742 (PV019367) and phage CC31 (NC 014662). More distantly related phages, including vB_Ecl_id4496 and SonariX, occupied separate branches, consistent with their lower nucleotide similarity. Intergenomic similarity was further evaluated using VIRIDIC, which performs whole‐genome comparisons in accordance with criteria established by the International Committee on Taxonomy of Viruses (https://ictv.global/). The resulting heatmap (Figure 4b) demonstrates high similarity values within the core clade (90%−95%), alongside consistently lower values for more distantly related phages. Of note, no pairwise comparison involving phage RZH exceeded the 95% nucleotide identity threshold used for species demarcation. The concordance between BLASTn similarity, VIRIDIC intergenomic comparisons, and phylogenetic indicates phage RZH is genomically distinct from all currently described species and can be considered as a novel species within this genus Karamvirus.

**Figure 3:**
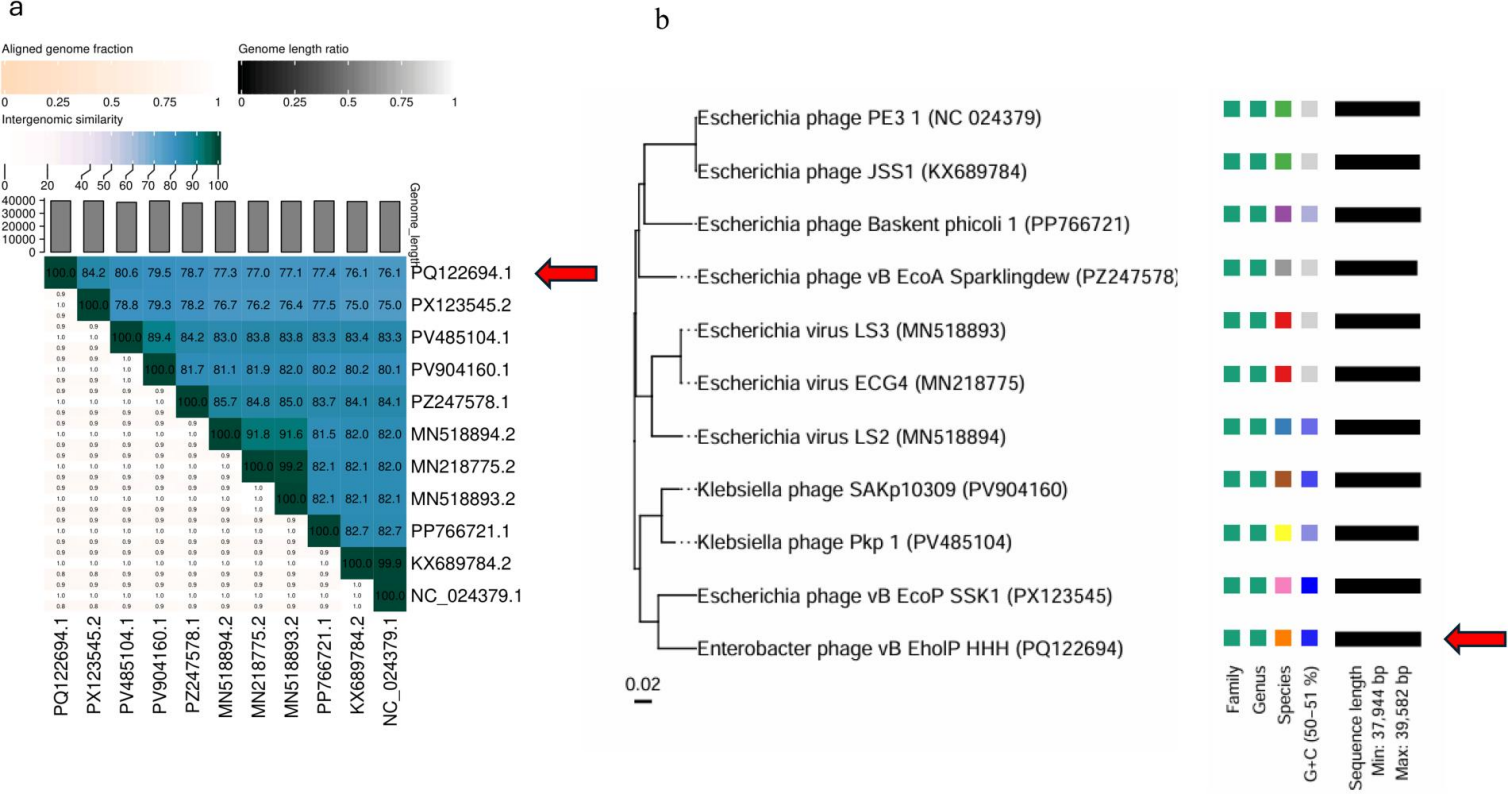
VIRIDIC and VICTOR analysis of Enterobacter phage vB_EhoIP_HHH. (a) VIRIDIC heat map showing pairwise intergenomic similarity between vB_EhoIP_HHH (PQ122694.1) and related reference phages. Similarity values aligned genome fraction and genome length ratio are shown to support genome‐based taxonomic comparison. (b) VICTOR whole‐genome phylogeny demonstrating that vB_EhoIP_HHH clusters among related Escherichia, Klebsiella and Enterobacter phages and forms a distinct sister branch to Escherichia phage vB_EcoP_SSK1 (PX123545). Associated metadata indicate predicted family, genus and species groupings, GC content, and genome length. The scale bar indicates substitutions per site.

**Figure 4:**
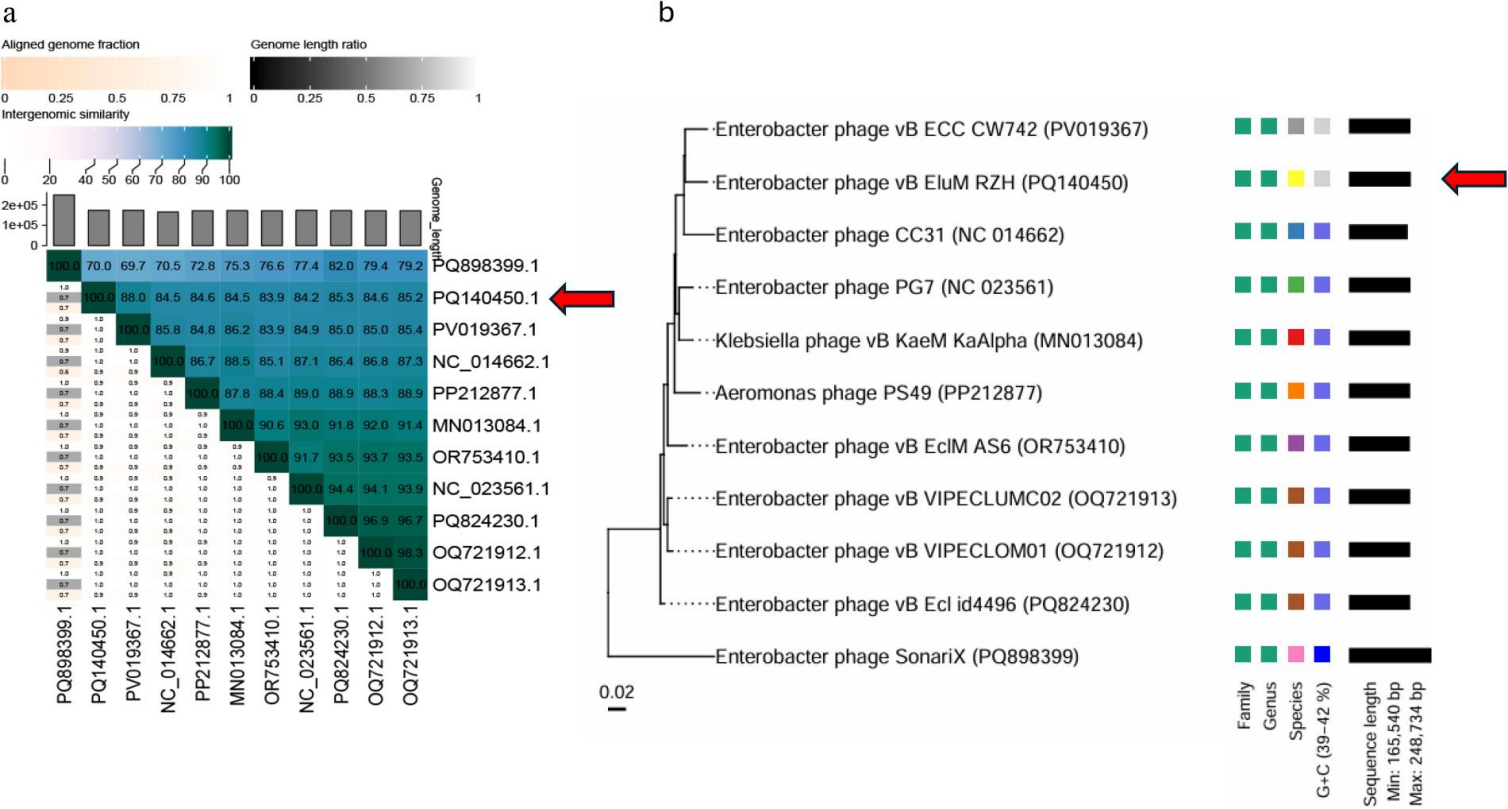
VIRIDIC and VICTOR analysis of Enterobacter phage vB_EluM_RZH. (a) VIRIDIC heat map showing pairwise intergenomic similarity between vB_EluM_RZH (PQ140450.1) and related reference phages. Similarity values aligned genome fraction and genome length ratio are shown to support genome‐based taxonomic comparison. (b) VICTOR whole‐genome phylogeny demonstrating that vB_EluM_RZH clusters closely with related Enterobacter phages, including vB_ECC_CW742 and CC31, while occupying a distinct branch within the clade.

BLASTn was used for comparative genomic analysis of phage _HHH, eleven closely related phages were selected based on nucleotide similarity. Among these, Escherichia phage PE3‐1 (NC_024379.1) exhibited the highest similarity to phage HHH, with 91.15% nucleotide identity and 83% query coverage, whereas Escherichia virus LS2 (MN518894.2) showed the lowest similarity, with 90.4% identity and 85% query coverage. Whole‐genome phylogenetic analysis by VICTOR (Figure 3b), placed phage HHH with *Enterobacter, Escherichia* and *Klebsiella*‐infecting phages, sharing a separate clade but with separate terminal branches with its closest relative Escherichia phage vB EcoP SSK1 (PX123545), indicating close evolutionary relatedness while remaining genomically distinct. These findings were further supported by VIRIDIC analysis (Figure 3a), which revealed intergenomic similarity values ranging from approximately 73%−84.2%, with the highest similarity observed with vB EcoP SSK1 (84.2%), supporting its classification as a novel species within the genus *Kayfunavirus* (Figure 4).

Host range analysis showed both phages had distinct but complementary lytic profiles across ECC and *E. coli* isolates. Phage HHH acted primarily against *E. hormaechei* isolates and a few ECC strains, whereas RZH displayed broader activity against *E. ludwigii* and ECC. Importantly, BCU‐17 was susceptible to both HHH and RZH and was therefore selected for subsequent biofilm checkerboard assays. This enabled the two phages to be tested in combination against the same MDR ECC biofilm‐forming strain, rather than evaluating each phage separately against its original propagation host. Both strains did demonstrate cross‐species lytic activity against *E. coli*, suggesting bacterial isolates may share common surface receptors (Sørensen et al. 2024). Furthermore, Efficiency of plating analysis supported the qualitative host‐range findings. vB_HHH showed the highest plating efficiency on its propagation host, *E. ludwigii BCU‐10*, with moderate EOP values across several *ECC* isolates, including BCU‐17. vB_RZH showed the highest plating efficiency on *E. hormaechei BCU‐9* and demonstrating infection on isolates BCU‐17.18 and BCU 25. Although EOP analysis provided quantitative evidence of productive infection across susceptible strains, the present study did not identify the bacterial receptors used by either phage or directly test depolymerase activity. These experiments would be valuable for future approaches to define molecular basis of host specificity and any enzyme‐mediated contribution to biofilm disruption (Tables 1).

**Table 1:** Host range and EOP of bacteriophages vB_EholP_HHH and vB_EluM_RZH across *Enterobacter cloacae complex (ECC) and Escherichia coli isolate*.

Bacterial strain	vB_HHH spot test (lysis +/‐)	vB_HHH EOP	vB_RZH spot test (lysis +/‐)	vB_RZH EOP	Interpretation
E.*hormaechei BCU‐9 (Host strain)*	—	ND	+	1.0 ± 0.01	Productive infection confirmed. Not susceptible to HHH
*E. hormaechei BCU‐25*	—	ND	+	0.5 ± 0.04	Susceptible to RZH only; moderate productive infection
*E. cloacae BCU‐11*	+	0.52 ± 0.05	—	ND	Susceptible to HHH only; moderate productive infection.
*E. cloacae BCU‐17*	+	0.67 ± 0.09	+	0.41 ± 0.05	Susceptible to both HHH and RZH; selected as shared host for cocktail biofilm assays.
*E. ludwigii BCU‐10 (host strain)*	+	1.0 ± 0.08	—	ND	HHH propagation host; productive infection confirmed. Not susceptible to RZH.
*E. ludwigii BCU‐22*	+	0.54 ± 0.08	—	ND	Susceptible to HHH only; moderate productive infection.
*ECC BCU‐18*	+	0.52 ± 0.03	+	0.43 ± 0.07	Susceptible to both HHH and RZH; supports complementary host range.
*ECC BCU‐6*	+	0.56 ± 0.04	—	ND	Susceptible to HHH only; moderate productive infection
*E. coli BCU‐1*	—	NT	—	NT	Not susceptible to either phage under spot‐test conditions
*E. coli BCU‐8*	—	NT	+	NT	Spot‐test positive for RZH only; EOP not determined
*E. coli BCU‐13*	—	NT	—	NT	Not susceptible to either phage under spot‐test conditions
*E. coli BCU‐15*	+	NT	—	NT	Spot‐test positive for HHH only; EOP not determined.

*Note:* E. hormaechei ‐ Enterobacter hormaechei E. ludwigii ‐ Enterobacter ludwigii, E. cloacae ‐ Enterobacter cloacae. ND—No plaques detected; NT—Not tested.

^1^ECC ‐ Enterobacter cloacae complex.

^I^dentified to complex level only. (+) clear zones, (‐) no lysis or plaque formation.

3.2

#### 3.2.1 Optimal Multiplicity of Infection (MOI), One‐Step Growth Curve and Adsorption Assay

**Figure 5:**
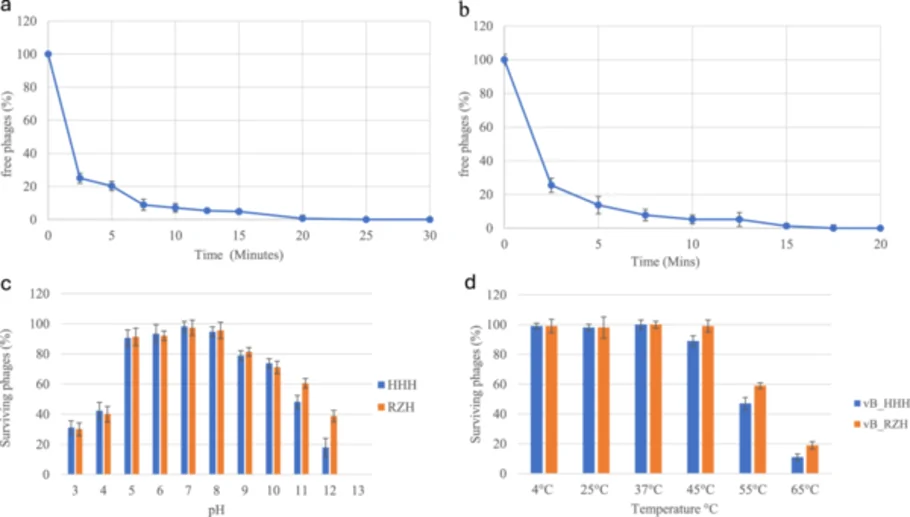
Adsorption kinetics and environmental stability of phages HHH and RZH. (a) Adsorption assay of phage HHH showing the percentage of free, unadsorbed phage particles over time following incubation with the bacterial host. HHH demonstrated rapid adsorption, with a sharp decline in free phage within the first 5–10 min and near‐complete adsorption by 20–25 min. (b) Adsorption assay of phage RZH showing a similar reduction in free phage particles over time, with rapid adsorption occurring during the early incubation period and minimal free phage remaining by 15–20 min. (c) pH stability of phages HHH and RZH following exposure to pH 3–12. Both phages remained highly stable across a broad pH range, particularly between pH 5 and pH 8, while reduced survival was observed under strongly acidic conditions and at alkaline pH 11–12. (d) Thermal stability of phages HHH and RZH following exposure to temperatures ranging from 4°C to 65°C. Both phages retained high viability at 4°C–37°C, with partial reduction at 45°C and marked loss of survival at 55°C and 65°C. Data are presented as mean ± SD.

MO1 0.01 achieved the highest phage titer with a value of 10^9^ PFU/mL for both phages. Phage HHH had a latent period of 24 min and a burst size of 60.8 virions per infected bacterial cell (Figure 1c). Whereas, phage RZH had a latent period of 30 min and a burst size of 73.5 virions per bacterial cell (Figure 1f). In terms of adsorption kinetics, phage HHH demonstrated, 95% of viral particles adsorbed to *E. ludwigii* within 10 min, with an adsorption constant *k*, of 1.5 × 10^−8 ^mL cell^−1^ min^−1^. (Figure 5a) Similarly, 93% of phage RZH had adsorbed to *E. hormaechi*, with an adsorption constant *k* of 1.3 × 10^−8 ^mL cell^−1^ min^−1^ (Figure 5b).

The high adsorption constants and the percentage of adsorbed viral particles for both phages indicate host recognition and rapid attachment. These properties allow efficient targeting of the bacteria and can reduce resistance development (Abedon 2023). Additionally, the shorter latent periods enable faster production of progeny, increasing the rate of amplification at the infection site, resulting in higher rates of bacterial clearance and reducing phage mutant resistance. Notably, there are differences, phage HHH displays a shorter latent period and smaller burst size, these differences can be attributed to infection strategies and the phage structure, which influence host interaction, replication dynamics and lytic efficacy (Dicks and Vermeulen 2024). Moreover, these values seem to be in a similar range reported for other Enterobacter phages (Subedi et al. 2025; Imanaka et al. 2025), however other Enterobacter phages demonstrated a stronger burst size (Chen et al. 2021).

#### 3.2.2 Temperature and pH on Phage Stability

Temperatures 4°C, 25°C, 37°C, 45°C, 55°C and 65°C were used to assess the thermal stability of the phage. Survival rates of both phages were between 98% − 100% at temperatures ranging from 4°C to 37°C, At 45°C, survival rate for phage HHH decreased to 88.7% (a 11.3% difference from 37°C), whereas phage RZH remained stable with a survival rate of 98.4%. There was a decline in survival rates for both phages at 55°C when compared to 37°C (58% and 41.3%, respectively). Survival rates at 65°C demonstrated a similar decline, with a loss of 87.6% and 79%, respectively, (Figure 5c,d). Influence of pH on both phages were assessed across a range of pH ranging from 3 to 12; At pH 3 both phages survival rates were low, indicating reduced inactivation, with phage HHH at 26.3% and phage RZH at 30%. At pH 4 both phages demonstrated partial tolerance to moderate acidic environments with survival rates at 42.% and 40%, respectively. However, between pH 5 and 8, both phages were relatively stable, and survival rates ranged from 90.5% to 94.8% for phage HHH and 91.3% to 95.6% for phage RZH. Optimum survival rates for both phages were at pH 7 (Phage HHH 98.2%; Phage RZH 97.4%). From pH 9, both phages started to show increasing sensitivity to alkaline conditions. When compared to pH 7, survival rates for phage HHH decreased between 19.3% and 83%, depending on pH and phage RZH activity decreased between 22.9% and 70% and both phages were completely inactivated at pH 13.

Temperature and pH can directly influence the physical and chemical stability of phages; Higher temperatures can accelerate degradation of proteins causing irreversible damage, whereas extreme pH conditions can cause the deamination of amino acids altering protein interactions (Ranveer et al. 2024). The results demonstrated both phages are stable under the conditions required for phage therapy or transport (Zhao et al. 2025), at temperatures ranging from 4°C to 37°C survival rates were 99%−100%, however from 45°C, viability of phages was reduced. Similarly, both phages were viable at pH levels 5–8, however at extreme acidic (3–4) and alkaline conditions (> 9) survival rates were reduced dramatically. These results are similar to other studies involving Enterobacter phages (González‐Gómez et al. 2024; Kuo et al. 2025). Notably, phage RZH demonstrated a slightly greater tolerance to temperature and alkaline conditions than phage HHH, which may be due to differences in capsid stability and phage morphology (Alič et al. 2017).

#### 3.2.3 Bacterial Lysis Assay

Lytic activity of both phages was determined through a standard lysis assay measuring the growth of the host bacterium at 0D_600 nm._ Both phages demonstrated lytic activity against their respective host, with slight differences. Phage HHH exhibited rapid lysis at higher MOIs (10–100), lysis occurred at more gradual level at MOI's 1−0.1, however OD values did begin to increase slightly after 200 min, indicating possible recovery of bacterial population via the emergence of resistant phage mutants. Phage RZH demonstrated a similar pattern, but lysis appeared uniform across all MOI's (Figure S6).

### 3.3 Biofilm Biomass Reduction Assay Using Phage Cocktail and EDTA

**Figure 6:**
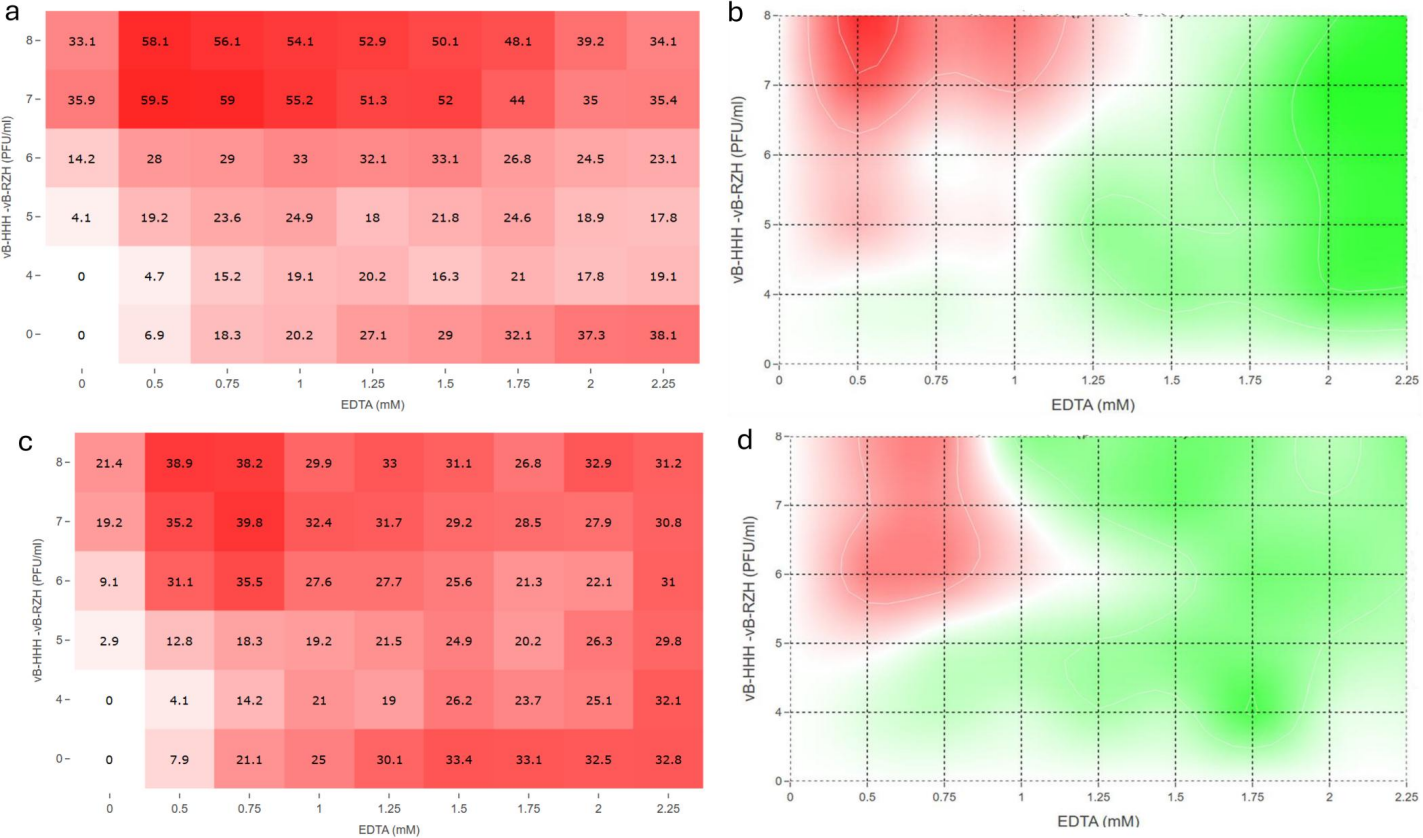
Antibiofilm activity and Bliss synergy analysis of the HHH/RZH phage cocktail combined with EDTA against MDR Enterobacter cloacae complex biofilms. (a) Percentage reduction in crystal violet‐stained biofilm biomass following treatment of 24 h biofilms with the HHH/RZH phage cocktail across increasing phage concentrations and EDTA concentrations. The greatest biomass reduction was observed at intermediate EDTA concentrations, particularly 0.5–1.0 mM EDTA in combination with higher phage concentrations. (b) Two‐dimensional Bliss synergy contour plot for the 24 h biofilm, showing the interaction between phage concentration and EDTA. Red regions indicate synergistic interactions, whereas green regions indicate antagonistic interactions. Synergy was concentrated around phage concentrations ≥ 10^7^ PFU/mL and EDTA concentrations of approximately 0.5–1.0 mM, while higher EDTA concentrations shifted the interaction towards additivity or antagonism. (c) Percentage reduction in biofilm biomass following treatment of mature 48 h biofilms with the HHH/RZH phage cocktail and EDTA. Compared with the 24 h biofilm, the 48‐h biofilm showed lower overall biomass reduction, indicating increased biofilm tolerance with maturation. The strongest reductions again occurred within the lower‐to‐intermediate EDTA range. (d) Two‐dimensional Bliss synergy contour plot for the 48‐h biofilm. The synergistic region was narrower than that observed in the 24 h biofilm and was primarily restricted to intermediate phage concentrations and EDTA concentrations below approximately 1.0 mM. Together, these data indicate that phage–EDTA antibiofilm activity is concentration‐dependent and most effective within a defined low‐millimolar EDTA window.

Antibiofilm activity of phages HHH and RZH in a phage cocktail in combination with EDTA was evaluated against MDR *Enterobacter cloacae* biofilms at 24 and 48 h using CV biomass assay. At 24 h, biomass reduction ranged between 0% and 59.5% depending on the treatment combination (Figure 6a). Greatest reduction was observed with phage MOI 10 (PFU/mlL 10^7^) and 0.5 mM EDTA, which reduced biomass by 59.5%, closely followed by MOI 10–0.75 mM EDTA (59%). However, combination efficacy declined at higher EDTA concentrations, particularly at ≥ 1.25 mM).

Bliss independence analysis of the 24 h biofilm (Figure 6b), revealed a localized region of synergy centered around phage MOI 10 and 0.5–1.0 mM EDTA. However, when the complete checkerboard matrix was analyzed, the overall interaction between EDTA and the phage cocktail was significantly antagonistic, with a mean Bliss score of −6.13 (*p* = 3.14 × 10^−3^). This global antagonism was driven primarily by higher EDTA concentrations, particularly ≥ 1.25 mM, where the interaction shifted towards additivity or antagonism despite some residual biomass reduction. Therefore, the 24 h biofilm data indicate a concentration‐dependent response, with localized synergy occurring only within a defined low‐millimolar EDTA window rather than across the full treatment matrix.

A similar pattern was observed in the 48‐h mature biofilm (Figure 6c,d). Localized synergy was again detected at intermediate EDTA concentrations, particularly around MOI 10 and 0.5–0.75 mM EDTA, but the synergistic region was narrower than that observed in the 24 h biofilm. Across the full checkerboard matrix, Bliss analysis indicated significant global antagonism, with a mean Bliss score of −7.72 (*p* = 1.17 × 10^−6^).

Crystal violet staining was used as a high‐throughput screening endpoint to quantify total attached biofilm biomass, including extracellular matrix material, dead cells and viable bacteria, across the phage–EDTA checkerboard. Therefore, a reduction in crystal violet staining was not interpreted as direct evidence of reduced viable bacterial burden. Instead, CFU enumeration was performed on selected optimal and comparator combinations to determine whether the treatment window identified by crystal violet staining and Bliss analysis corresponded to reduced bacterial recovery. These data supported the biomass and Bliss findings, showing that MOI 10 combined with 0.75–1.0 mM EDTA reduced the recovery of viable bacteria, particularly in 24 h biofilms. However, CFU enumeration was not performed across the full checkerboard matrix; therefore, the checkerboard data should be interpreted primarily as a biomass and interaction screen, with CFU enumeration providing targeted viability confirmation.

**Figure 7:**
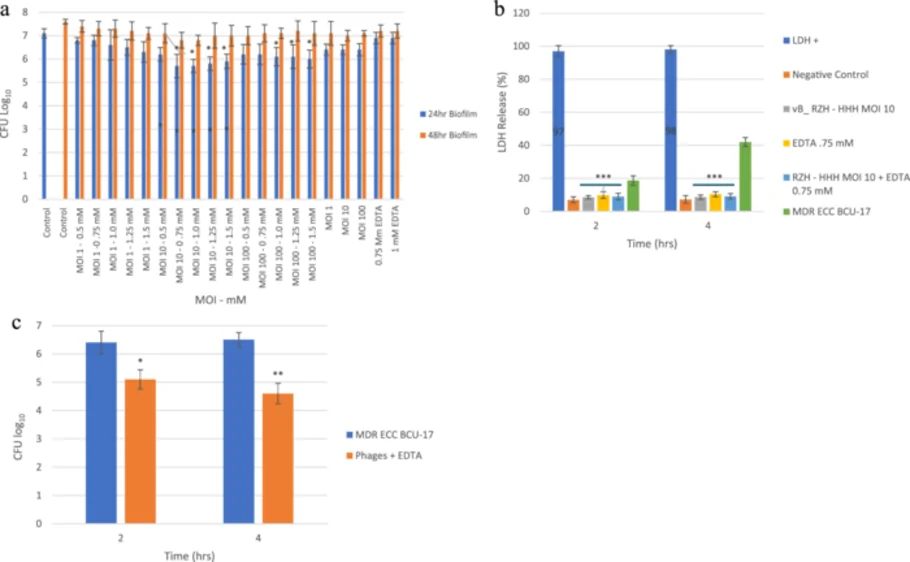
Viable biofilm burden, cytotoxicity and fibroblast infection response following HHH/RZH phage–EDTA treatment. (a) Log_10_ CFU recovery from 24 and 48 h MDR Enterobacter cloacae complex biofilms following treatment with selected HHH/RZH phage cocktail and EDTA combinations. The greatest viable count reduction was observed with MOI 10 + 0.75–1.0 mM EDTA, with 24 h biofilms showing greater susceptibility than 48 h biofilms. (b) LDH release assay showing mammalian cell membrane damage following exposure to phage cocktail, EDTA, phage–EDTA combination or E. cloacae. Phage MOI 10, 0.75 mM EDTA and MOI 10 + 0.75 mM EDTA produced low LDH release, whereas E. cloacae induced time‐dependent cytotoxicity. (c) CFU recovery from fibroblast infection assays. Treatment with HHH/RZH MOI 10 + 0.75 mM EDTA significantly reduced recoverable *E. cloacae* at 2 and 4 h compared with infected untreated controls. Data are presented as mean ± SD; asterisks indicate statistically significant differences compared with the relevant control.

CFU enumeration was performed to determine whether the biomass reduction observed by crystal violet staining translated into a reduction of recoverable viable bacteria. In the 24 h biofilm, combinations of MOI 10 with 0.75–1.0 mM EDTA produced the lowest bacterial burden, reducing counts to approximately 5.6 log_10_ CFU, corresponding to a 1.4 log_10_ reduction compared with the untreated control (7.0 log_10_ CFU; *p* < 0.005). The combination of MOI 10 with 1.25 mM EDTA produced a similar but slightly lower reduction of 1.3 log_10_ CFU (Figure 7a). By comparison, in the 48‐h biofilm, the greatest CFU reduction was also observed with MOI 10 and 0.75–1.0 mM EDTA, reducing bacterial recovery to approximately 6.8 log_10_ CFU, equivalent to a 0.8 log_10_ reduction compared with the untreated control (7.6 log_10_ CFU). Phage‐only and EDTA‐only controls produced more modest reductions in recoverable bacteria than the corresponding combination treatments, suggesting that antibiofilm activity was enhanced by the complementary action of phage and EDTA. Mechanistically, EDTA may disrupt the EPS matrix by chelating divalent cations, particularly calcium (Ca^2+^) and magnesium (Mg^2+^), which contribute to biofilm stability. This may increase matrix porosity and facilitate phage penetration into deeper regions of the biofilm (Ferriol‐González and Domingo‐Calap 2020). EDTA can also destabilize the lipopolysaccharide layer of Gram‐negative bacteria by removing divalent cations, thereby increasing outer‐membrane permeability and potentially facilitating phage receptor access and bacterial lysis (Duc et al. 2023; Liu et al. 2022). However, Bliss analysis of both 24 h and 48 h biofilms showed that synergy was restricted to a narrow EDTA concentration window, with reduced synergy above 1.25–1.5 mM and clear antagonistic interactions at 2.25 mM. This suggests a biphasic EDTA effect: at lower concentrations, EDTA may enhance antibiofilm activity by weakening matrix and outer‐membrane barriers, whereas at higher concentrations, excessive chelation may interfere with phage stability or phage–host interactions (Ma et al. 2018). This interpretation is consistent with previous work showing that low‐millimolar EDTA concentrations can enhance phage‐mediated inhibition by suppressing bacterial regrowth (Huang et al. 2021). It is also supported by Ma et al. (2018), who demonstrated that bacteriophage killing of extraintestinal pathogenic *Escherichia coli* was strongly influenced by metal availability. In that study, EDTA inhibited phage‐mediated killing, whereas supplementation with exogenous calcium, magnesium and iron restored or enhanced phage activity, indicating that free metal ion availability can influence phage efficacy and that excessive EDTA‐mediated chelation may impair phage–host interactions.

Within the data, MOI 10 had a greater efficacy than MOI 100, suggesting that higher phage concentration does not necessarily result in lower biomass, as seen in other studies (Wu et al. 2024; Singh et al. 2025; Lin et al. 2025). This may be multifactorial, such as host‐depletion trade off where an overabundance of phages results in lack of active hosts to infect leading to reduced propagation and amplification (Urgeya et al. 2025). Notably, when there are too many phages present at the same time, multiple phages will attempt to infect a single bacterium, which can lead to “lysis from without” (Namonyo et al. 2023) reducing phage progeny production. These findings seem to be consistent with a cation‐dependent interaction as described by Ma et al. 2018 (Ma et al. 2018), however the present study did not directly test whether EDTA‐mediated Ca^2+^/Mg^2+^ chelation altered phage adsorption, infection efficiency or phage stability within the biofilm matrix. Of note, the observed loss of synergy at higher EDTA concentrations should be interpreted as a biofilm‐level interaction rather than direct proof of impaired phage adsorption. However, these findings align with other studies showing that phage antibiofilm activity can be improved by adjunctive compounds that destabilize the biofilm matrix, increase bacterial susceptibility, or modify phage–host interactions. Direct studies examining phage–EDTA combinations against bacterial biofilms remain limited. However one relevant example was the use of bacteriophage P22 with EDTA and nisin against *Salmonella Typhimurium* biofilms (Yüksel et al. 2018), where combination treatment reduced biofilm formation and partially removed mature biofilms. However, this was a different Gram‐negative organism and involved a three‐component treatment system, rather than a defined phage cocktail combined with EDTA against MDR ECC biofilms. The present study therefore adds to the limited literature by showing that EDTA does not function as a uniformly beneficial phage adjunct. Instead, enhanced antibiofilm activity was restricted to a narrow low‐millimolar EDTA window, while higher EDTA concentrations reduced combination efficacy. This concentration‐dependent pattern is biologically plausible, as shown in a study (Ma et al. 2018) where EDTA‐mediated depletion of free metal ions can inhibit phage‐mediated bacterial killing, whereas supplementation with calcium, magnesium or iron restored phage activity. Although their study was performed in human blood rather than biofilms, it supports the broader principle that cation availability can influence phage performance. In the context of the present biofilm assay, this may help explain why lower EDTA concentrations enhanced phage‐mediated biofilm disruption, whereas excessive chelation at higher EDTA concentrations shifted the interaction towards antagonism.

A limitation of the antibiofilm analysis is that the phage–EDTA checkerboard assay was performed using a single shared susceptible ECC strain, BCU‐17. This design was selected to allow direct evaluation of the HHH/RZH cocktail against one reproducible biofilm‐forming host. Therefore, the EDTA‐dependent synergy and antagonism observed in this study should be interpreted as specific to BCU‐17 under the experimental conditions tested. However, future work should test the cocktail against a broader panel of MDR *Enterobacter* biofilm‐forming isolates to determine whether the observed EDTA‐dependent antibiofilm window is conserved across the wider ECC. Furthermore, Because the EDTA–phage interaction was measured in established biofilms rather than planktonic culture, the observed antagonism at higher EDTA concentrations should be interpreted as a biofilm‐level interaction. Future work will examine EDTA‐mediated effects within the biofilm matrix, including EPS disruption, divalent‐cation availability, phage penetration and phage recovery from treated biofilms. Calcium and magnesium supplementation within the biofilm assay would also help determine whether cation availability contributes to the observed shift from synergy to antagonism.

LDH release was used to assess mammalian cell membrane damage following exposure to the phage cocktail, EDTA and the optimized phage–EDTA combination. Treatment with the HHH/RZH phage cocktail at MOI 10, 0.75 mM EDTA, or the MOI 10 + 0.75 mM EDTA combination produced low LDH release at both 2 and 4 h, with values comparable to the negative control and significantly lower than the bacteria‐infected positive control (*p* < 0.001), indicating minimal membrane damage under the tested conditions (Figure 7b).

Bacteria from infected fibroblast culture supernatants was quantified following infection with MDR *E. cloacae* BCU‐17 and treatment with the optimized phage–EDTA combination. In untreated infected fibroblast cultures, bacterial counts remained high over the experimental period, reaching approximately 6.4 log_10_ CFU at 2 h and 6.5 log_10_ CFU at 4 h (Figure 7c).

Treatment with the HHH/RZH phage cocktail at MOI 10 combined with 0.75 mM EDTA reduced viable bacterial load at both timepoints. At 2 h, bacterial burden was reduced to approximately 5.1 log_10_ CFU, corresponding to 1.3 log_10_ CFU reduction compared with the untreated infected control. By 4 h, the combination reduced bacterial density to approximately 4.6 log_10_ CFU, representing a 1.9 log_10_ CFU reduction compared with the untreated infected control. The biofilm and fibroblast infection assays represent distinct biological environments and should not be directly compared. However, they provide complementary evidence supporting MOI 10 + 0.75 mM EDTA as the most promising treatment condition. In the biofilm model, this combination produced the greatest reduction in biomass and viable biofilm‐associated bacteria, whereas in the fibroblast model it reduced recoverable *E. cloacae* while maintaining low LDH release. Together, these findings indicate that the MOI 10 + 0.75 mM EDTA condition, selected from the biofilm checkerboard assay, reduced recoverable extracellular bacteria in the fibroblast infection model while maintaining low short‐term LDH release. However, as CFU enumeration in this model was performed using culture supernatants, this data reflects recoverable extracellular bacterial burden and not adherent, fibroblast‐associated or intracellular bacteria. Additionally, this assay was not designed to determine additivity or synergy in host‐cell culture.

## 4 Conclusion

This study identifies vB_EhoIP_HHH and vB_EluM_RZH as two novel lytic Enterobacter phages with genomic and phenotypic features that support their further investigation as antibiofilm candidates. When applied as a cocktail with EDTA, activity against MDR *Enterobacter cloacae* complex biofilm was strongly concentration dependent. The most effective response occurred within a narrow low‐millimolar EDTA window, particularly at MOI 10 combined with 0.5–0.75 mM EDTA, where both biofilm biomass and recoverable viable bacteria were reduced. Bliss independence analysis demonstrated that EDTA did not uniformly enhance phage activity; instead, synergy was localized to defined treatment conditions, while higher EDTA concentrations shifted the interaction towards antagonism. The optimized MOI 10 + 0.75 mM EDTA condition also reduced recoverable bacteria in the fibroblast infection model while maintaining low LDH release, supporting its further assessment in wound‐relevant systems. From a translational perspective, these findings suggest that EDTA may act as a dose‐sensitive adjunct rather than a universally beneficial enhancer of phage activity. This has implications for the development of phage‐based antibiofilm formulations for wound‐associated or device‐associated *Enterobacter* biofilms, where EDTA concentration may determine whether the interaction is beneficial or antagonistic. However, the underlying mechanism was not directly resolved within the biofilm matrix. Future work should examine EDTA‐mediated effects on EPS structure, divalent‐cation availability, phage penetration, phage recovery from treated biofilms and Ca^2+^/Mg^2+^ rescue under high‐EDTA conditions to determine how matrix disruption and phage activity interact within established ECC biofilms.

## Author Contributions

**Kashif Haq:** conceptualization, investigation, methodology, formal analysis, writing – original draft, writing – review and editing. **Zac Newland‐Smith:** formal analysis. **Martin Figgit:** conceptualization, supervision, writing – original draft. **David Lee:** supervision. **Kwabena Duedu:** supervision, conceptualization.

## Funding

The authors have nothing to report.

## Ethics Statement

The authors have nothing to report.

## Conflicts of Interest

The authors declare no conflicts of interest.

## Supporting information


Supporting File 1

## Data Availability

The complete genome sequences of Enterobacter phages vB_EhoIP_HHH and vB_EluM_RZH are available in GenBank under accession numbers PQ122694.1 and PQ140450.1, respectively. Bacterial genome data are available under BioProject accession numbers PRJNA1129045 and PRJNA1354818, with associated SRA metadata SUB14455581 and SUB15423642. Additional data supporting the findings of this study are provided in the Supplementary Information.
