# Supplementary material for: Genomic and Phenotypic Characterization of Two Novel Enterobacter Phages With EDTA‐Enhanced Antibiofilm Activity
Source: Microbiologyopen. 2026 Sep 2;15(5):e70396. doi: 10.1002/mbo3.70396 (PMC13538957; doi:10.1002/mbo3.70396)
Supplement: Supplementary file 1 [file MBO3-15-e70396-s001.docx]

Supplementary Data

Table S1 -S3 Antimicrobial susceptibility profile of *Enterobacter ludwigii* *Enterobacter .hormaechei*. MIC values were determined using VITEK 2 or manual E-test methods. Susceptibility interpretation was assigned according to clinical breakpoint guidance. MIC, minimum inhibitory concentration.

Table S1

| Organism | Antibiotic | MIC* | Profile |
| --- | --- | --- | --- |
| *E.ludwigii* | Amoxicillin | >32 | Resistant |
|  | Piperacillin / Tazobactam | >128 | Resistant |
|  | Cefuroxime | >64 | Resistant |
|  | Cefuroxime Axetil | >64 | Resistant |
|  | Cefotaxime | >64 | Resistant |
|  | Ceftazidime | >64 | Resistant |
|  | Cefepime | >64 | Resistant |
|  | Ertapenem | >8 | Resistant |
|  | Meropenem | >16 | Resistant |
|  | Amikacin | < 2 | Sensitive |
|  | Gentamicin | < 1 | Sensitive |
|  | Tobramycin | < 1 | Sensitive |
|  | Ciprofloxacin | < .25 | Sensitive |
|  | Tigecycline | 1 | Sensitive |
|  | Trimethoprim | < 20 | Sensitive |

Table S2

| Organism | Antibiotic | MIC* | Profile |
| --- | --- | --- | --- |
| *E.hormaechei* | Amoxicillin | >32 | Resistant |
|  | Piperacillin / Tazobactam | 8 | Resistant |
|  | Cefuroxime | >64 | Resistant |
|  | Cefuroxime Axetil | >64 | Resistant |
|  | Cefotaxime | >64 | Resistant |
|  | Ceftazidime | >64 | Resistant |
|  | Cefepime | >32 | Resistant |
|  | Ertapenem | >8 | Resistant |
|  | Meropenem | >16 | Resistant |
|  | Amikacin | < 2 | Sensitive |
|  | Gentamicin | 4 | Resistant |
|  | Tobramycin | 8 | Resistant |
|  | Ciprofloxacin | < .25 | Sensitive |
|  | Tigecycline | 1 | Sensitive |
|  | Trimethoprim | < 20 | Sensitive |

Table S3

| Organism | Antibiotic | MIC* | Profile |
| --- | --- | --- | --- |
| *E.cloacae* | Amoxicillin | >64 | Resistant |
|  | Piperacillin / Tazobactam | 8 | Resistant |
|  | Cefuroxime | >64 | Resistant |
|  | Cefuroxime Axetil | >64 | Resistant |
|  | Cefotaxime | >64 | Resistant |
|  | Ceftazidime | >64 | Resistant |
|  | Cefepime | >32 | Resistant |
|  | Ertapenem | >8 | Resistant |
|  | Meropenem | >16 | Resistant |
|  | Amikacin | < 2 | Sensitive |
|  | Gentamicin | 4 | Resistant |
|  | Tobramycin | 8 | Resistant |
|  | Ciprofloxacin | < .25 | Sensitive |
|  | Tigecycline | 1 | Sensitive |
|  | Trimethoprim | >320 | Resistant |

Table S3 and S4. Predicted antimicrobial resistance-associated genes and mechanisms identified in *E.ludwigii* and *E.hormaechei* using the PATRIC Genome Annotation Service.

AMR-associated genes were predicted using the PATRIC k-mer-based detection method, which compares genome sequences against a curated collection of representative AMR gene sequence variants. Predicted genes are grouped according to their assigned resistance mechanism, including antibiotic inactivation, target protection or replacement, efflux-mediated resistance, permeability modulation, altered cell wall charge, and regulation of resistance-associated gene expression. These annotations indicate the presence of multiple intrinsic and acquired resistance-associated determinants within the E. ludwigii genome and support its multidrug-resistant profile

Table S4

| AMR Mechanism | Genes |
| --- | --- |
| Antibiotic activation enzyme | KatG |
| Antibiotic inactivation enzyme | ACT/MIR family, bla_ACT-12_ |
| Antibiotic resistance gene cluster, cassette, or operon | MarA, MarB, MarR |
| Antibiotic target in susceptible species | Alr, Ddl, dxr, EF-G, EF-Tu, folA, Dfr, folP, gyrA, gyrB, Iso-tRNA, kasA, MurA, rho, rpoB, rpoC, S10p, S12p |
| Antibiotic target protection protein | BcrC |
| Efflux pump conferring antibiotic resistance | AcrAB-TolC, AcrAD-TolC, AcrEF-TolC , AcrZ, EmrAB-TolC, EmrD, MacA, MacB, MdfA/Cmr, MdtABC-TolC, MdtL, SugE, TolC/OpmH |
| Gene conferring resistance via absence | gidB |
| Protein altering cell wall charge conferring resistance | GdpD, PgsA |
| Protein modulating permeability | OccD6/OprQ |
| Regulator modulating expression of antibiotic resistance genes | AcrAB-TolC, EmrAB-TolC, H-NS, OxyR |

Table S5

| AMR Mechanism | Genes |
| --- | --- |
| Antibiotic activation enzyme | KatG |
| Antibiotic inactivation enzyme | ACT/MIR family, APH(3'')-I, APH(6)-Ic/APH(6)-Id, CatA1/CatA4 family, KPC family, TEM family |
| Antibiotic resistance gene cluster, cassette, or operon | MarA, MarB, MarR |
| Antibiotic target in susceptible species | Alr, Ddl, dxr, EF-G, EF-Tu, folA, Dfr, folP, gyrA, gyrB, Iso-tRNA, kasA, MurA, rho, rpoB, rpoC, S10p, S12p |
| Antibiotic target protection protein | BcrC |
| Efflux pump conferring antibiotic resistance | AcrAB-TolC, AcrAD-TolC, AcrEF-TolC , AcrZ, EmrAB-TolC, EmrD, MacA, MacB, MdfA/Cmr, MdtABC-TolC, MdtL, SugE, TolC/OpmH |
| Gene conferring resistance via absence | gidB |
| Protein altering cell wall charge conferring resistance | GdpD, PgsA |
| Protein modulating permeability | OccD6/OprQ |
| Regulator modulating expression of antibiotic resistance genes | AcrAB-TolC, EmrAB-TolC, H-NS, OxyR |
|  | |

Table S6. Predicted coding sequences and functional module annotation of Enterobacter phage vB_EhoIP_HHH.

Predicted coding sequences (CDSs) within the 39,582 bp genome of phage vB_EhoIP_HHH are listed according to genomic position, strand orientation, predicted product and assigned functional module. Functional annotations were generated using automated genome annotation pipelines and refined by manual BLASTp-based comparison against conserved protein databases. CDSs were grouped into putative modules associated with DNA packaging, replication and metabolism, lysis, and structural assembly. Hypothetical proteins represent predicted ORFs with no confidently assigned function based on current database matches.

| CDS | Start | End | Strand | Product | Module |
| --- | --- | --- | --- | --- | --- |
| 1 | 1 | 264 | Positive | terminase small subunit | Packaging |
| 2 | 371 | 820 | Positive | endopeptidase Rz | Lysis |
| 3 | 850 | 2580 | Positive | terminase large subunit | Packaging |
| 4 | 2621 | 2728 | Positive | hypothetical protein |  |
| 5 | 2725 | 2835 | Positive | hypothetical protein |  |
| 6 | 2871 | 3029 | Positive | hypothetical protein |  |
| 7 | 3196 | 3285 | Negative | hypothetical protein |  |
| 8 | 3299 | 3394 | Negative | hypothetical protein |  |
| 9 | 3482 | 3598 | Negative | hypothetical protein |  |
| 10 | 3756 | 3872 | Negative | hypothetical protein |  |
| 11 | 4011 | 4199 | Negative | hypothetical protein |  |
| 12 | 4193 | 4306 | Negative | hypothetical protein |  |
| 13 | 4328 | 4504 | Positive | hypothetical protein |  |
| 14 | 4568 | 4756 | Positive | hypothetical protein |  |
| 15 | 4769 | 5293 | Positive | hypothetical protein |  |
| 16 | 5287 | 5442 | Positive | hypothetical protein |  |
| 17 | 5445 | 5570 | Positive | hypothetical protein |  |
| 18 | 5640 | 5744 | Positive | hypothetical protein |  |
| 19 | 5744 | 5938 | Positive | hypothetical protein |  |
| 20 | 5938 | 6264 | Positive | hypothetical protein |  |
| 21 | 6361 | 9042 | Positive | DNA-directed RNA polymerase | Replication |
| 22 | 9055 | 9255 | Positive | hypothetical protein |  |
| 23 | 9334 | 9801 | Positive | hypothetical protein |  |
| 24 | 9892 | 10071 | Positive | hypothetical protein |  |
| 25 | 10075 | 10335 | Positive | hypothetical protein |  |
| 26 | 10341 | 11372 | Positive | ATP-dependent DNA ligase | Replication |
| 27 | 11434 | 11529 | Positive | hypothetical protein |  |
| 28 | 11576 | 11836 | Positive | hypothetical protein |  |
| 29 | 11829 | 12254 | Positive | nucleotide kinase | Replication |
| 30 | 12244 | 12339 | Positive | hypothetical protein |  |
| 31 | 12336 | 12500 | Positive | host RNA polymerase inhibitor | Replication |
| 32 | 12548 | 13246 | Positive | ssDNA binding protein | Replication |
| 33 | 13282 | 13704 | Positive | endonuclease |  |
| 34 | 13698 | 14168 | Positive | hypothetical protein |  |
| 35 | 14158 | 14616 | Positive | endolysin amidase | Lysis |
| 36 | 14631 | 14843 | Positive | hypothetical protein |  |
| 37 | 14912 | 16615 | Positive | DNA primase/helicase | Replication |
| 38 | 16660 | 16824 | Positive | hypothetical protein |  |
| 39 | 16896 | 19067 | Positive | DNA polymerase | Replication |
| 40 | 19067 | 19270 | Positive | hypothetical protein |  |
| 41 | 19282 | 19566 | Positive | hypothetical protein |  |
| 42 | 19563 | 19772 | Positive | hypothetical protein |  |
| 43 | 19769 | 20044 | Positive | hypothetical protein |  |
| 44 | 20037 | 20903 | Positive | exonuclease | Replication |
| 45 | 20885 | 20977 | Positive | hypothetical protein |  |
| 46 | 21114 | 21386 | Positive | hypothetical protein |  |
| 47 | 21401 | 21625 | Positive | DUF5476 family protein | Replication / lysis |
| 48 | 21630 | 22034 | Positive | "putative acetyltransferase | Replication |
| 49 | 22027 | 22275 | Positive | hypothetical protein |  |
| 50 | 22287 | 23855 | Positive | collar head-to-tail connector protein | Structural |
| 51 | 23963 | 24847 | Positive | capsid and scaffold | Structural |
| 52 | 24876 | 24971 | Negative | hypothetical protein |  |
| 53 | 24986 | 25981 | Negative | hypothetical protein |  |
| 54 | 25956 | 26051 | Positive | hypothetical protein |  |
| 55 | 26098 | 26304 | Positive | hypothetical protein |  |
| 56 | 26362 | 26928 | Positive | tail fiber protein | Structural |
| 57 | 26928 | 27155 | Positive | RNA binding protein | Replication |
| 58 | 27165 | 29522 | Positive | non-contractile tail tubular protein | Structural |
| 59 | 29599 | 30069 | Positive | internal virion protein |  |
| 60 | 30054 | 30641 | Positive | internal virion protein |  |
| 61 | 30653 | 32935 | Positive | internal virion protein |  |
| 62 | 32941 | 36837 | Positive | virion protein | Lysis |
| 63 | 36903 | 39362 | Positive | hypothetical protein |  |

Table S7. Predicted coding sequences and functional module annotation of Enterobacter phage vB_EluM_RZH.

Predicted coding sequences (CDSs) within the 39,582 bp genome of phage vB_EhoIP_HHH are listed according to genomic position, strand orientation, predicted product and assigned functional module. Functional annotations were generated using automated genome annotation pipelines and refined by manual BLASTp-based comparison against conserved protein databases. CDSs were grouped into putative modules associated with DNA packaging, replication and metabolism, lysis, and structural assembly. Hypothetical proteins represent predicted ORFs with no confidently assigned function based on current database matches

| CDS | Start | End | Strand | Product | Role |
| --- | --- | --- | --- | --- | --- |
| 1 | 1108 | 1343 | + | rIIA protein | regulation or lysis |
| 2 | 1336 | 2274 | + | rIIB lysis inhibitor | Lysis |
| 3 | 2315 | 2656 | + | hypothetical protein |  |
| 4 | 2649 | 2978 |  | hypothetical protein |  |
| 5 | 3535 | 3654 | + | hypothetical protein |  |
| 6 | 3665 | 3871 | + | hypothetical protein |  |
| 7 | 3927 | 4373 | + | nucleoid disruption protein | Replication |
| 8 | 4375 | 4557 | + | hypothetical protein |  |
| 9 | 4554 | 4688 | + | hypothetical protein |  |
| 10 | 4695 | 6074 | + | DNA topoisomerase | Replication |
| 11 | 6261 | 6524 | + | hypothetical protein |  |
| 12 | 6532 | 6804 | + | hypothetical protein |  |
| 13 | 6914 | 7558 | + | transcriptional regulator of middle promoters | Replication |
| 14 | 7573 | 7983 | + | hypothetical protein |  |
| 15 | 7980 | 8363 | + | hypothetical protein |  |
| 16 | 8363 | 8560 | + | hypothetical protein |  |
| 17 | 8580 | 8669 | + | hypothetical protein |  |
| 18 | 8653 | 8877 | + | hypothetical protein |  |
| 19 | 8952 | 9230 | + | hypothetical protein |  |
| 0 | 9227 | 9577 | + | hypothetical protein |  |
| 21 | 9577 | 9837 | + | hypothetical protein |  |
| 22 | 9905 | 10177 | + | "anti-sigma factor | Replication |
| 23 | 10186 | 10842 | - | Holin | Lysis |
| 24 | 10872 | 11660 | - | long tail fiber distal subunit | Structural |
| 25 | 11691 | 14261 | - | hypothetical protein |  |
| 26 | 14269 | 14922 | - | long tail fiber protein | Structural |
| 27 | 14983 | 16134 | - | long tail fiber proximal connector | Structural |
| 28 | 16134 | 19928 | - | long tail fiber proximal subunit | Structural |
| 29 | 19998 | 20921 | + | ribonuclease H | Replication |
| 30 | 20929 | 21204 | + | dsDNA binding protein | Replication |
| 31 | 21182 | 21481 | + | late promoter transcription accessory protein" | Replication |
| 32 | 21478 | 22131 | + | DNA helicase loader | Replication |
| 33 | 22142 | 23062 | + | single stranded DNA-binding protein | Replication |
| 34 | 23098 | 23262 | + | hypothetical protein |  |
| 35 | 23231 | 23452 | + | hypothetical protein |  |
| 36 | 23492 | 23806 | + | hypothetical protein |  |
| 37 | 23838 | 24086 | + | hypothetical protein |  |
| 38 | 24146 | 24430 | + | hypothetical protein |  |
| 39 | 24430 | 24756 | + | hypothetical protein |  |
| 40 | 24737 | 24835 | + | hypothetical protein |  |
| 41 | 24835 | 25074 | + | hypothetical protein |  |
| 42 | 25074 | 25286 | + | hypothetical protein |  |
| 43 | 25286 | 25879 | + | dihydrofolate reductase | replication |
| 44 | 25866 | 26189 | + | hypothetical protein |  |
| 45 | 26176 | 26523 | + | hypothetical protein |  |
| 46 | 26523 | 27383 | + | thymidylate synthase | replication |
| 47 | 27380 | 27568 | + | dTMP thymidylate synthase | Replication |
| 48 | 27568 | 27795 | + | hypothetical protein |  |
| 49 | 27774 | 28103 | + | hypothetical protein |  |
| 50 | 28090 | 30345 | + | "NrdA-like aerobic NDP reductase large subunit | Replcation |
| 51 | 30396 | 30602 | + | hypothetical protein |  |
| 52 | 30599 | 30817 | + | hypothetical protein |  |
| 53 | 30814 | 32028 | + | ribonucleotide-diphosphate reductase subunit beta" | Replcation |
| 54 | 32055 | 32468 | + | endonuclease | Replcation |
| 55 | 32489 | 33595 | + | RNA ligase | Replcation |
| 56 | 33658 | 34164 | + | inhibitor of host transcription | Replcation |
| 57 | 34512 | 34508 | + | spanin inner membrane subunit | Lysis |
| 58 | 34505 | 34807 | + | hypothetical protein |  |
| 59 | 34821 | 34925 | + | hypothetical protein |  |
| 60 | 34936 | 35415 | + | hypothetical protein |  |
| 61 | 35412 | 35627 | + | hypothetical protein |  |
| 62 | 35617 | 35955 | + | hypothetical protein |  |
| 63 | 35952 | 36125 | + | hypothetical protein |  |
| 64 | 36136 | 36405 | + | 3'-phosphatase 5'-polynucleotide kinase | Replcation |
| 65 | 36433 | 37008 | + | polynucleotide kinase | Replication |
| 66 | 37008 | 37199 | + | hypothetical protein |  |
| 67 | 37213 | 37431 | + | hypothetical protein |  |
| 68 | 37431 | 37724 | + | hypothetical protein |  |
| 69 | 37714 | 37944 | + | hypothetical protein |  |
| 70 | 37944 | 38288 | + | hypothetical protein |  |
| 71 | 38288 | 38854 | + | dCMP deaminase | Replication |
| 72 | 38820 | 39161 | + | hypothetical protein |  |
| 73 | 39154 | 39483 | + | hypothetical protein |  |
| 74 | 39516 | 39863 | + | head assembly chaperone protein | Structural |
| 75 | 39989 | 40243 | + | lysis inhibition accessory protein | Lysis |
| 76 | 40392 | 40571 | + | hypothetical protein |  |
| 77 | 40624 | 40827 | + | hypothetical protein |  |
| 78 | 40896 | 41270 | + | hypothetical protein |  |
| 79 | 41295 | 41828 | + | hypothetical protein |  |
| 80 | 41900 | 42262 | + | hypothetical protein |  |
| 81 | 42301 | 42771 | + | hypothetical protein |  |
| 82 | 42771 | 42938 | + | hypothetical protein |  |
| 83 | 42271 | 42938 | + | hypothetical protein |  |
| 84 | 42935 | 43138 | + | hypothetical protein |  |
| 85 | 43215 | 43307 | + | hypothetical protein |  |
| 86 | 43286 | 43462 | + | hypothetical protein |  |
| 87 | 43452 | 44315 | + | hypothetical protein |  |
| 88 | 44312 | 44542 | + | hypothetical protein |  |
| 89 | 44601 | 46061 | + | DNA ligase | Replication |
| 90 | 46058 | 46276 | + | hypothetical protein |  |
| 91 | 46331 | 48466 | + | NAD(+)--arginine ADP-ribosyltransferase | Replication |
| 92 | 48526 | 48816 | + | hypothetical protein |  |
| 93 | 48843 | 49805 | - | tail assembly protein | Structural |
| 94 | 49805 | 50851 | - | baseplate tail tube cap | Structural |
| 95 | 50861 | 52621 | - | baseplate hub subunit and tail length | Structural |
| 96 | 52618 | 53145 | - | baseplate hub assembly protein | Structural |
| 97 | 53078 | 54262 | - | baseplate hub subunit | Structural |
| 98 | 54259 | 55011 | - | baseplate hub assembly protein | Structural |
| 99 | 55061 | 55690 | + | baseplate hub | Structural |
| 100 | 55690 | 56088 | + | baseplate wedge subunit | Structural |
| 101 | 56164 | 56577 | + | single stranded DNA-binding protein | Replication |
| 102 | 56577 | 56762 | + | hypothetical protein |  |
| 103 | 56793 | 56960 | + | hypothetical protein |  |
| 104 | 57020 | 57250 | - | DNA helicase | Replication |
| 105 | 57258 | 58760 | - | DNA helicase |  |
| 106 | 58813 | 59535 | + | protein Inh | Regulation |
| 107 | 59569 | 60126 | + | capsid and scaffold protein | Structural |
| 108 | 60107 | 60202 | + | hypothetical protein |  |
| 109 | 60202 | 60444 | + | hypothetical protein |  |
| 110 | 60428 | 60781 | + | hypothetical protein |  |
| 111 | 60896 | 61144 | + | hypothetical protein |  |
| 112 | 61153 | 62157 | + | RNA ligase | Replication |
| 113 | 62157 | 62702 | + | DprA-like DNA recombination-mediator protein | Replication |
| 114 | 62735 | 64018 | - | head vertex protein | Structural |
| 115 | 64104 | 65666 | - | major capsid protein | Structural |
| 116 | 65688 | 66503 | - | capsid assembly scaffolding protein | Structural |
| 117 | 66537 | 67178 | - | prohead core protein protease | Structural |
| 118 | 67178 | 67603 | - | head scaffolding protein | Structural |
| 119 | 67613 | 67855 | - | prohead core protein | Structural |
| 120 | 67855 | 69423 | - | portal protein | Structural |
| 121 | 69484 | 69975 | - | tail protein | Structural |
| 122 | 69993 | 70112 | - | hypothetical protein |  |
| 123 | 70093 | 72075 | - | tail sheath protein | Structural |
| 124 | 72109 | 73941 | - | terminase large subunit | Packaging |
| 125 | 73925 | 74422 | - | terminase small subunit | Packaging |
| 126 | 74422 | 75252 | - | tail sheath stabilizer and completion protein | Structural |
| 127 | 75298 | 76098 | - | neck protein | Structural |
| 128 | 76095 | 77036 | - | neck protein | Structural |
| 129 | 77067 | 78479 | - | fibritin neck whisker | Structural |
| 130 | 78488 | 80038 | - | short tail fiber protein | Structural |
| 131 | 80038 | 80700 | - | baseplate wedge subunit and tail pin | Structural |
| 132 | 80700 | 82508 | - | baseplate wedge protein | Structural |
| 133 | 82505 | 83413 | - | baseplate protein | Structural |
| 134 | 83475 | 84551 | - | baseplate wedge protein | Structural |
| 135 | 84478 | 87576 | - | baseplate wedge subunit | Structural |
| 136 | 87573 | 89519 | - | baseplate wedge subunit | Structural |
| 137 | 89524 | 89820 | - | hypothetical protein |  |
| 138 | 89820 | 90398 | - | putative homing endonuclease | Replication |
| 139 | 90379 | 90882 | - | hypothetical protein |  |
| 140 | 90890 | 92620 | - | baseplate central spike complex protein | Structural |
| 141 | 92620 | 93195 | - | baseplate wedge subunit | Structural |
| 142 | 93245 | 93694 | + | head completion protein | Structural |
| 143 | 93682 | 94338 | + | homing endonuclease | Replication |
| 144 | 94338 | 95168 | + | DNA end protector protein | Replication |
| 145 | 95250 | 95480 | + | tail completion protein | Structural |
| 146 | 95842 | 96570 | + | deoxynucleoside monophosphate kinase | Replication |
| 147 | 96567 | 96821 | + | tail fiber chaperone | Structural |
| 148 | 96818 | 97276 | + | hypothetical protein |  |
| 149 | 97361 | 97540 | + | hypothetical protein |  |
| 150 | 97527 | 97727 | + | hypothetical protein |  |
| 151 | 97711 | 98004 | + | hypothetical protein |  |
| 152 | 97970 | 98329 | + | hypothetical protein |  |
| 153 | 98842 | 99000 | + | hypothetical protein |  |
| 154 | 99170 | 99262 | + | hypothetical protein |  |
| 155 | 99704 | 99889 | - | hypothetical protein |  |
| 156 | 100269 | 100493 | + | hypothetical protein |  |
| 157 | 100610 | 100984 | + | hypothetical protein |  |
| 158 | 100995 | 101492 | + | hypothetical protein |  |
| 159 | 101492 | 101710 | + | hypothetical protein |  |
| 160 | 101688 | 101834 | + | hypothetical protein |  |
| 161 | 101834 | 102283 | + | hypothetical protein |  |
| 162 | 102287 | 102511 | + | hypothetical protein |  |
| 163 | 103004 | 103204 | + | hypothetical protein |  |
| 164 | 103290 | 103538 | + | hypothetical protein |  |
| 165 | 103661 | 103921 | + | hypothetical protein |  |
| 166 | 103946 | 104194 | + | hypothetical protein |  |
| 167 | 104249 | 104851 | + | hypothetical protein |  |
| 168 | 104905 | 105558 | + | hypothetical protein |  |
| 169 | 105497 | 105835 | + | hypothetical protein |  |
| 170 | 105844 | 106077 | + | hypothetical protein |  |
| 171 | 106131 | 106472 | + | hypothetical protein |  |
| 172 | 106502 | 107419 | + | hypothetical protein |  |
| 173 | 107453 | 107827 | + | hypothetical protein |  |
| 174 | 107830 | 108186 | + | hypothetical protein |  |
| 175 | 108170 | 108511 | + | hypothetical protein |  |
| 176 | 108504 | 108866 | + | hypothetical protein |  |
| 177 | 108863 | 109174 | + | hypothetical protein |  |
| 178 | 109171 | 109401 | + | hypothetical protein |  |
| 179 | 109464 | 109877 | + | Nudix hydrolase | Replication |
| 180 | 109911 | 110405 | + | baseplate hub + tail lysozyme | Structural |
| 181 | 110392 | 110685 | + | hypothetical protein |  |
| 182 | 110745 | 111152 | + | endonuclease | Replication |
| 183 | 111174 | 111302 | - | hypothetical protein |  |
| 184 | 111330 | 111884 | + | hypothetical protein |  |
| 185 | 112069 | 112308 | + | hypothetical protein |  |
| 186 | 112298 | 112462 | + | hypothetical protein |  |
| 187 | 112459 | 112737 | + | hypothetical protein |  |
| 188 | 112737 | 112949 | + | hypothetical protein |  |
| 189 | 112949 | 113179 | + | hypothetical protein |  |
| 190 | 113238 | 113702 | + | endonuclease | Replication |
| 191 | 113723 | 114271 | + | hypothetical protein |  |
| 192 | 114271 | 114597 | + | valyl-tRNA synthetase modifie | Replication |
| 193 | 114594 | 115013 | + | hypothetical protein |  |
| 194 | 115010 | 115474 | + | hypothetical protein |  |
| 195 | 115474 | 116058 | + | thymidine kinase | Replication |
| 196 | 116068 | 116232 | + | hypothetical protein |  |
| 197 | 116229 | 116360 | + | hypothetical protein |  |
| 198 | 116417 | 166776 | + | hypothetical protein |  |
| 199 | 116836 | 117048 | + | hypothetical protein |  |
| 200 | 117056 | 117331 | + | lysis inhibition regulator membrane protein | Lysis |
| 201 | 117387 | 117707 | + | starvation-inducible transcriptional regulator | regulation |
| 202 | 117815 | 118273 | + | hypothetical protein |  |
| 203 | 118273 | 118380 | + | hypothetical protein |  |
| 204 | 118380 | 118649 | + | DksA-like zinc-finger protein | regulation |
| 205 | 118652 | 118939 | + | hypothetical protein |  |
| 206 | 118936 | 119142 | + | hypothetical protein |  |
| 207 | 119126 | 119404 | + | hypothetical protein |  |
| 208 | 119397 | 119642 | + | hypothetical protein |  |
| 209 | 119668 | 120120 | + | membrane protein | Lysis |
| 210 | 120120 | 120341 | + | hypothetical protein |  |
| 211 | 120351 | 120899 | + | hypothetical protein |  |
| 212 | 120896 | 121321 | + | hypothetical protein |  |
| 213 | 121318 | 122328 | + | hypothetical protein |  |
| 214 | 122331 | 122585 | + | hypothetical protein |  |
| 215 | 122551 | 122649 | + | hypothetical protein |  |
| 216 | 122704 | 122988 | + | hypothetical protein |  |
| 217 | 123070 | 123312 | + | hypothetical protein |  |
| 218 | 123386 | 124363 | + | hypothetical protein |  |
| 219 | 124400 | 124741 | + | hypothetical protein |  |
| 220 | 124752 | 125255 | + | hypothetical protein |  |
| 221 | 125252 | 125725 | + | hypothetical protein |  |
| 222 | 125712 | 125912 | + | hypothetical protein |  |
| 223 | 125912 | 126121 | + | hypothetical protein |  |
| 224 | 126121 | 126537 | + | hypothetical protein |  |
| 225 | 126534 | 126902 | + | hypothetical protein |  |
| 226 | 127068 | 127835 | + | hypothetical protein |  |
| 227 | 127835 | 128041 | + | hypothetical protein |  |
| 228 | 128041 | 129108 | + | hypothetical protein |  |
| 229 | 129105 | 129362 | + | hypothetical protein |  |
| 230 | 129359 | 129787 | + | hypothetical protein |  |
| 231 | 129800 | 130315 | + | hypothetical protein |  |
| 232 | 130312 | 130647 | + | hypothetical protein |  |
| 233 | 130644 | 130937 | + | hypothetical protein |  |
| 234 | 130934 | 131227 | + | hypothetical protein |  |
| 235 | 131187 | 131468 | + | hypothetical protein |  |
| 236 | 131465 | 131638 | + | hypothetical protein |  |
| 237 | 131638 | 131949 | + | hypothetical protein |  |
| 238 | 131934 | 132080 | + | hypothetical protein |  |
| 239 | 132064 | 132522 | + | PinA-like inhibitor of host protease | regulation |
| 240 | 132522 | 132686 | + | hypothetical protein |  |
| 241 | 132720 | 133193 | + | recombinase endonuclease | Replication |
| 242 | 133190 | 135019 | + | ribonucleotide reductase of class III (anaerobic) large subunit | Replication |
| 243 | 135009 | 135494 | + | anaerobic ribonucleotide reductase small subunit | Replication |
| 244 | 135478 | 135585 | + | hypothetical protein |  |
| 245 | 135594 | 135869 | + | hypothetical protein |  |
| 246 | 135844 | 136158 | + | glutaredoxin | Replication |
| 247 | 136195 | 136332 | + | hypothetical protein |  |
| 248 | 136332 | 136603 | + | hypothetical protein |  |
| 249 | 136608 | 136874 | + | hypothetical protein |  |
| 250 | 136940 | 137278 | + | hypothetical protein |  |
| 251 | 137275 | 137589 | + | hypothetical protein |  |
| 252 | 137586 | 137849 | + | hypothetical protein |  |
| 253 | 137842 | 138087 | + | hypothetical protein |  |
| 254 | 138080 | 138307 | + | hypothetical protein |  |
| 255 | 138304 | 138555 | + | hypothetical protein |  |
| 256 | 138552 | 138848 | + | hypothetical protein |  |
| 257 | 138915 | 139448 | + | RNA polymerase sigma factor for late transcription | Regulation |
| 258 | 139432 | 139659 | + | hypothetical protein |  |
| 259 | 139665 | 139988 | + | hypothetical protein |  |
| 260 | 140163 | 140411 | + | hypothetical protein |  |
| 261 | 140519 | 141541 | + | SbcD-like subunit of palindrome specific- endonuclease | Replication |
| 262 | 141538 | 141762 | + | hypothetical protein |  |
| 263 | 141759 | 143447 | + | recombination-related endonuclease | Replication |
| 264 | 143444 | 143635 | + | hypothetical protein |  |
| 265 | 143645 | 144055 | + | RNA polymerase binding protein | Regulation |
| 266 | 144075 | 144761 | + | putative sliding clamp | Replication |
| 267 | 144821 | 145780 | + | clamp loader small subunit | Replication |
| 268 | 145777 | 146340 | + | gp62; DNA polymerase accessory protein | Replication |
| 269 | 146342 | 146707 | + | endoribonuclease translational repressor | Regulation |
| 270 | 146707 | 146868 | + | hypothetical protein |  |
| 271 | 146966 | 149674 | + | DNA polymerase | Replication |
| 272 | 149735 | 150175 | + | hypothetical protein |  |
| 273 | 150172 | 150912 | + | thymidylate synthase | Replication |
| 274 | 150903 | 151739 | + | glucosyl transferase | Replication / Metabolism |
| 275 | 151736 | 152812 | + | beta glucosyl transferase | Replication / Metabolism |
| 276 | 152916 | 154082 | + | hypothetical protein |  |
| 277 | 154051 | 154440 | + | hypothetical protein |  |
| 278 | 154517 | 155893 | + | DNA primase/helicase | Replication |
| 279 | 155955 | 156236 | + | hypothetical protein |  |
| 280 | 156246 | 156236 | + | hypothetical protein |  |
| 281 | 156246 | 156527 | + | hypothetical protein |  |
| 282 | 156524 | 156715 | + | hypothetical protein |  |
| 283 | 156703 | 157038 | + | hypothetical protein |  |
| 284 | 157035 | 157223 | + | hypothetical protein |  |
| 285 | 157239 | 157706 | + | hypothetical protein |  |
| 286 | 157746 | 158771 | + | DNA primase subunit |  |
| 287 | 158768 | 159052 | + | hypothetical protein |  |
| 288 | 159098 | 159616 | + | dUTPase | Replication |
| 289 | 160009 | 160248 | + | virion structural protein | Structural |
| 290 | 160329 | 160580 | - | hypothetical protein |  |
| 291 | 160580 | 160972 | + | hypothetical protein |  |
| 292 | 160965 | 161474 | + | hypothetical protein |  |
| 293 | 161489 | 161680 | + | hypothetical protein |  |
| 294 | 161741 | 162418 | + | RNA polymerase ADP-ribosylase | regulation |
| 295 | 162593 | 163255 | + | Srd anti-sigma factor |  |
| 296 | 163255 | 163569 | + | hypothetical protein |  |
| 297 | 163596 | 164909 | + | DNA helicase | Replication |
| 298 | 164915 | 165157 | + | hypothetical protein |  |
| 299 | 165154 | 165843 | + | exodeoxyribonuclease | Replication |
| 300 | 165883 | 166350 | - | hypothetical protein |  |
| 301 | 166417 | 166635 | + | modifier of suppressor tRNAs | Replication |
| 302 | 166635 | 167312 | + | hypothetical protein |  |
| 303 | 167300 | 167647 | + | hypothetical protein |  |
| 304 | 167634 | 167810 | + | FmdB-like transcriptional regulator | regulation |
| 305 | 167810 | 167905 | + | hypothetical protein |  |
| 306 | 167902 | 168162 | + | hypothetical protein |  |
| 307 | 168162 | 168371 | + | hypothetical protein |  |
| 308 | 168403 | 169938 | + | head outer capsid protein | Structural |
| 309 | 169938 | 170045 | + | hypothetical protein |  |
| 310 | 170017 | 170403 | + | hypothetical protein |  |
| 311 | 170449 | 172293 | + | DNA topoisomerase large subunit | Replication |
| 312 | 172333 | 172488 | + | hypothetical protein |  |
| 313 | 172534 | 172818 | + | hypothetical protein |  |
| 314 | 172812 | 173018 | + | hypothetical protein |  |

Fig S1. (a) TOPCONS consensus topology prediction for ORF73, showing no predicted transmembrane helices or signal peptide across the analysed protein sequence. The consensus prediction classifies the protein as non-membrane associated. (b) DeepTMHMM topology prediction showing ORF73 as a globular protein, with posterior probability supporting an intracellular/non-transmembrane localisation and no membrane-spanning regions. (c) SignalP 6.0 prediction showing no detectable signal peptide, with the sequence classified as “other”. Together, these analyses indicate that ORF73 lacks predicted transmembrane domains and a classical secretion signal, supporting its annotation as a soluble hypothetical protein and a candidate lysis-associated protein requiring further functional validation

Figure S2 Predicted membrane topology and signal peptide analysis of ORF59 from phage HHH.

(a)TOPCONS consensus topology prediction for ORF59, showing no predicted transmembrane helices or signal peptide across the analysed protein sequence. The consensus prediction supports a non-membrane-associated topology. (b) DeepTMHMM topology prediction classifying ORF59 as a globular protein (c) SignalP 6.0 prediction showing no detectable signal peptide, with the sequence classified as “other

Figure S3. Predicted membrane topology and signal peptide analysis of ORF60 from phage HHH.

(a)TOPCONS consensus topology prediction for ORF60, showing no predicted transmembrane helices across the protein sequence, although an N-terminal signal peptide region is predicted by several topology algorithms. (b) DeepTMHMM topology prediction classifying ORF60 as a globular protein with no membrane-spanning regions. (c) SignalP 6.0 prediction indicating the presence of an N-terminal Sec/SPI signal peptide, with a predicted cleavage site around the N-terminal region.

Figure S4. Predicted membrane topology and signal peptide analysis of ORF61 from phage HHH.

(a)TOPCONS consensus topology prediction for ORF61, showing no predicted transmembrane helices or signal peptide across the analysed protein sequence. The consensus prediction indicates a non-membrane-associated topology. (b) DeepTMHMM topology prediction classifying ORF61 as a globular protein (c) SignalP 6.0 prediction showing no detectable signal peptide, with the sequence classified as “other”

Figure S5. Predicted membrane topology and signal peptide analysis of ORF62 from phage HHH.

(a) TOPCONS consensus topology prediction for ORF62, showing no predicted transmembrane helices or signal peptide across the analysed protein sequence. The consensus prediction supports a predominantly non-membrane-associated topology. (b) DeepTMHMM topology prediction classifying ORF62 as a globular protein with posterior probability supporting an outside/non-transmembrane localisation and no membrane-spanning regions. (c) SignalP 6.0 prediction showing no detectable signal peptide, with the sequence classified as “other”.

Figure S6. Bacterial lysis activity of phages HHH and RZH against their respective Enterobacter hosts.

(a) Lysis kinetics of Enterobacter ludwigii following treatment with phage HHH at MOIs 100, 10, 1, 0.1 and 0.01. Bacterial growth was monitored by measuring absorbance at 600 nm over 480 min. Phage-treated cultures showed rapid suppression of bacterial growth compared with the untreated control, with higher MOIs producing sustained inhibition. (b) Lysis kinetics of Enterobacter hormaechei following treatment with phage RZH across the same MOI range. RZH reduced bacterial growth across all tested MOIs, with strong early lytic activity and sustained suppression relative to the untreated control. Data are presented as mean ± SD from three independent biological replicates.
